# High-fat diet exacerbates cognitive decline in mouse models of Alzheimer's disease and mixed dementia in a sex-dependent manner

**DOI:** 10.1186/s12974-022-02466-2

**Published:** 2022-05-14

**Authors:** Olivia J. Gannon, Lisa S. Robison, Abigail E. Salinero, Charly Abi-Ghanem, Febronia M. Mansour, Richard D. Kelly, Alvira Tyagi, Rebekah R. Brawley, Jordan D. Ogg, Kristen L. Zuloaga

**Affiliations:** 1 Department of Neuroscience & Experimental Therapeutics, Albany Medical College, 47 New Scotland Avenue; MC-136, Albany, NY 12208 USA; 2 Department of Psychology & Neuroscience, Nova Southeastern University, 3301 College Avenue, Fort Lauderdale, FL 33314 USA; 3 Department of Psychology, Stetson University, 421 N Woodland Blvd, DeLand, FL 32723 USA

**Keywords:** Sex, Vascular, Dementia, Alzheimer’s disease, High-fat diet, Inflammation, Obesity, Cerebral hypoperfusion, Metabolic, Glucose intolerance, Diabetes

## Abstract

**Background:**

Approximately 70% of Alzheimer’s disease (AD) patients have co-morbid vascular contributions to cognitive impairment and dementia (VCID); this highly prevalent overlap of dementia subtypes is known as mixed dementia (MxD). AD is more prevalent in women, while VCID is slightly more prevalent in men. Sex differences in risk factors may contribute to sex differences in dementia subtypes. Unlike metabolically healthy women, diabetic women are more likely to develop VCID than diabetic men. Prediabetes is 3× more prevalent than diabetes and is linked to earlier onset of dementia in women, but not men. How prediabetes influences underlying pathology and cognitive outcomes across different dementia subtypes is unknown. To fill this gap in knowledge, we investigated the impact of diet-induced prediabetes and biological sex on cognitive function and neuropathology in mouse models of AD and MxD.

**Methods:**

Male and female 3xTg-AD mice received a sham (AD model) or unilateral common carotid artery occlusion surgery to induce chronic cerebral hypoperfusion (MxD model). Mice were fed a control or high fat (HF; 60% fat) diet from 3 to 7 months of age. In both sexes, HF diet elicited a prediabetic phenotype (impaired glucose tolerance) and weight gain.

**Results:**

In females, but not males, metabolic consequences of a HF diet were more severe in AD or MxD mice compared to WT. In both sexes, HF-fed AD or MxD mice displayed deficits in spatial memory in the Morris water maze (MWM). In females, but not males, HF-fed AD and MxD mice also displayed impaired spatial learning in the MWM. In females, but not males, AD or MxD caused deficits in activities of daily living, regardless of diet. Astrogliosis was more severe in AD and MxD females compared to males. Further, AD/MxD females had more amyloid beta plaques and hippocampal levels of insoluble amyloid beta 40 and 42 than AD/MxD males. In females, but not males, more severe glucose intolerance (prediabetes) was correlated with increased hippocampal microgliosis.

**Conclusions:**

High-fat diet had a wider array of metabolic, cognitive, and neuropathological consequences in AD and MxD females compared to males. These findings shed light on potential underlying mechanisms by which prediabetes may lead to earlier dementia onset in women.

**Supplementary Information:**

The online version contains supplementary material available at 10.1186/s12974-022-02466-2.

## Background

Diabetes increases the risk of developing dementia by twofold [1–5]. While diabetes is increasingly common, prediabetes is estimated to affect 1 out of every 3 Americans [6], and most people are unaware of their status. Like diabetes, prediabetes is characterized by impaired glucose tolerance; however, those with prediabetes show slight elevations in insulin and fasting blood glucose, rather than hyperglycemia. Diabetes and prediabetes are shared risk factors and common co-morbidities for the two most common forms of dementia: Alzheimer’s disease (AD) and vascular contributions to cognitive impairment and dementia (VCID) [1–5]. Obesity, which is often co-morbid with prediabetes or type 2 diabetes, increases AD risk threefold and VCID fivefold [7–9]. AD is characterized by amyloid plaques, tau tangles, and neurodegeneration culminating in brain atrophy and cognitive impairment. VCID is caused by deficits in cerebral blood flow and/or damage to cerebral vessels. In reality, the distinction between AD and VCID is less clear-cut. Dementia pathologies often overlap, with more than half of dementia patients having multiple pathologies [10], a condition known as mixed dementia (MxD). The most common form of MxD is a mix of AD and VCID, as this occurs in ~ 70% of AD patients [11–13]. MxD is underrepresented in animal research despite its high clinical prevalence. Understanding the interaction between AD and VCID risk factors will provide insight into MxD.

Dementia risk and prevalence vary by sex: women are more likely to develop AD[10], while men are slightly more likely to develop VCID [14, 15]. Discrepancies in risk factors may drive these sex differences. In patients with diabetes, the sex difference observed in the non-diabetic population is reversed: diabetic women are at a 19% greater risk of VCID than diabetic men [3]. Among those who have VCID, women are also more likely to have diabetes [16]. Prediabetes is associated with cognitive impairment and earlier onset of dementia in women, but not men, suggesting it may be a sex-specific risk factor [17]. However, it is unknown how prediabetes affects MxD, and whether these effects differ by sex.

High-fat (HF) diet is commonly used to induce metabolic disease in rodents, as it causes both obesity and prediabetes. HF diet can have profound effects on the brain, some of which are sex dependent. For example, we have shown that HF diet impairs adult hippocampal neurogenesis in female, but not male mice [18]. Further, we recently found that HF diet in middle-aged mice causes a wider array of cognitive deficits in females compared to males [19]. Others have found that a HF diet in the 3xTg-AD model of AD exacerbates cognitive impairment [20–25] and AD pathology, such as brain atrophy [20], inflammation [22, 26], and Aβ load [26–28]. Examination of sex differences in the cognitive effects of HF diet in the 3xTg-AD mouse have been mixed, with some studies finding greater cognitive impairment in females [20], while others have found no sex differences [21]. Previously, we reported that HF diet results in greater metabolic impairment (weight gain, visceral fat, and glucose intolerance) in female compared to male 3xTg-AD mice [29]. HF-fed females also showed increased astrogliosis in the hypothalamus, a brain region that controls metabolic function. Whether these greater metabolic disturbances in females would also lead to more severe cognitive deficits and neuropathology in brain regions associated with learning and memory had not yet been tested. In the current study, using mouse models of both AD and MxD, we found that diet-induced obesity with prediabetes led to a wider array of cognitive deficits and neuropathology in females compared to males.

## Methods

### Animals and experimental design

**Fig. 1:**
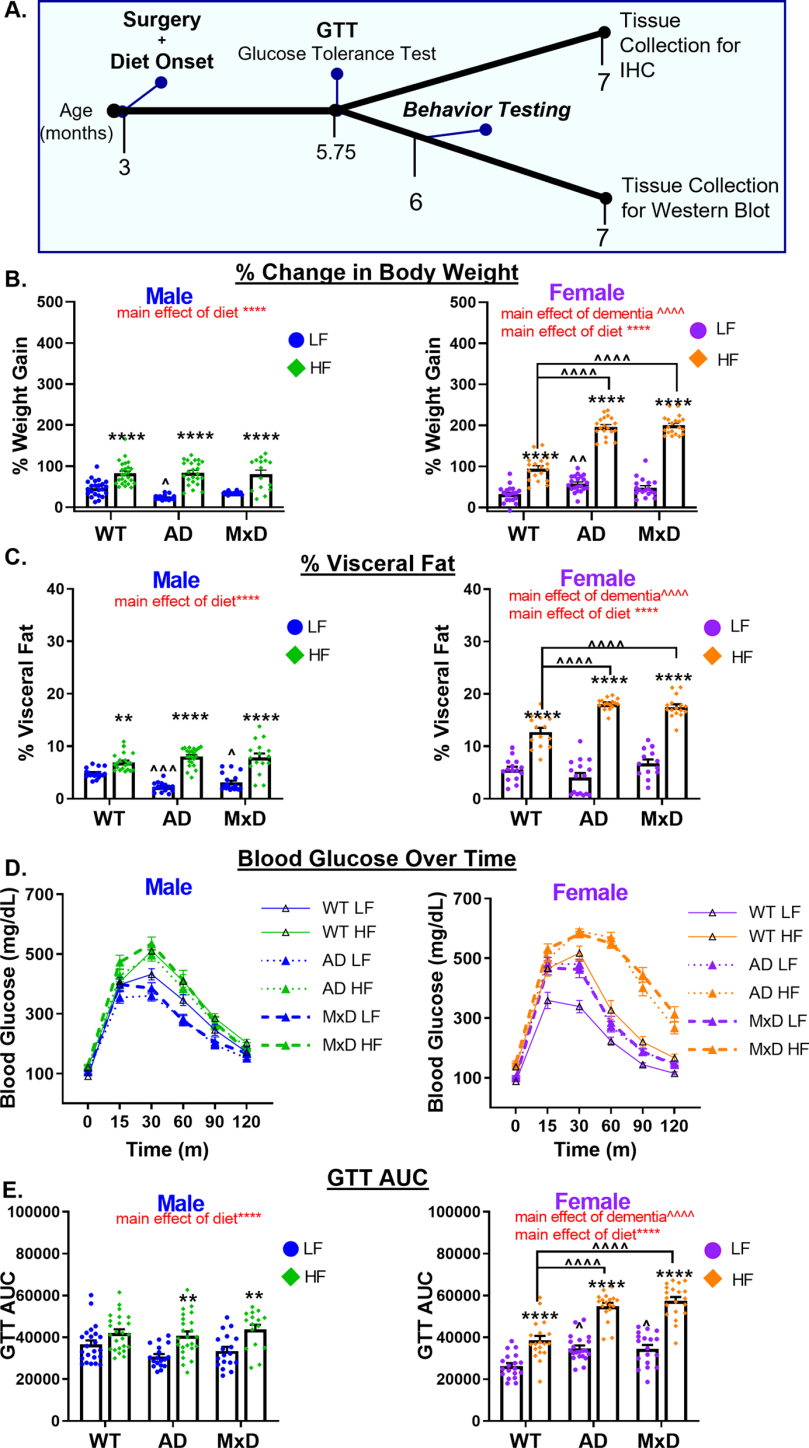
HF diet caused greater metabolic impairment in AD and MxD females compared to males. **A** Experimental timeline. GTT (glucose tolerance test). **B** Weight gain was assessed by the % change in body weight from the start of the study to the end of the study. **C** Visceral adiposity was determined by isolating and weighing the visceral fat pads and normalizing to body weight. **D**, **E** Glucose intolerance was assessed with a GTT following a 16 h fast. **D** Glucose clearance was gauged by concentrations of glucose in the blood measured over time (time 0 = fasting blood glucose). **E** Blood glucose concentration over time was used to calculate area under the curve. We previously reported metabolic data for the Sham WT and Sham AD, but not the MxD, groups in Robison et al. (2020) in the *Journal of Neuroinflammation* [29]; licensed under a Creative Commons Attribution 4.0 International License; (https://creativecommons.org/licenses/by/4.0/). Data are presented as mean + SEM, **p < 0.01 effect of diet, ****p < 0.0001 effect of diet, ^*p* < 0.05 effect of dementia, ^^^*p* < 0.001 effect of dementia, ^^^^*p* < 0.0001 effect of dementia, 2-way ANOVA, (*n* = 13–25/group)

This study was conducted in accordance with the National Institutes of Health Guidelines for the Care and Use of Laboratory Animals, and protocols were approved by the Institutional Animal Care and Use Committee at Albany Medical College (Albany, NY, USA). Temperature and humidity were set at 72 °F, 30–70% humidity, with a 12-h light/dark cycle (7 a.m. on/7 p.m. off). Mice were fed a standard chow diet (Purina Lab Diet 5P76) until the start of this study. They were housed in Allentown cages at a density of 2–5 mice. Mice were provided with environmental enrichment (Nestlets and Shepherd Shacks) and were group housed at all times, except during the nest building test. Male and female wild-type (WT) B6129SF2/J mice (#101045) and 3xTg-AD (#34830-JAX) breeding pairs were purchased from Jackson Laboratories (Bar Harbor, ME) and used to maintain a colony at Albany Medical Center’s Animal Resource Facility. The 3xTg-AD mice, which are on a C57BL/6;129X1/SvJ;129S1/Sv background, have three mutations that are associated with AD in humans: APPSwe, tauP301L, and Psen1^tm1Mpm^ [30]. A timeline of the experiment is shown in Fig. 1A. At ~ 3 months of age, 3xTg-AD mice underwent a sham surgery (AD group) or a unilateral common carotid artery occlusion surgery (MxD group). WT controls also received a sham surgery (WT group). One week following surgery, mice were placed on either a HF diet (60% fat, 5.24 kcal/g; D12492, Research Diets, New Brunswick, NJ) or a low-fat (LF) control diet (10% fat, 3.82 kcal/g; D12450B, Research Diets) for the duration of the study. At ~ 5.75 months of age, mice underwent a glucose tolerance test (GTT) and a 2-week rest period, behavioral testing, followed by blood flow imaging, euthanasia, and tissue collection (including brains, fat, and reproductive organs) at ~ 7 months of age. A total of 251 mice were used in this study. Experiments were conducted in cohorts of up to 20 animals. A subset of mice (n = 118) was designated for collection of metabolic data, behavior testing, plasma assays [reported in [29]], and blood flow imaging, and brains were microdissected for use in Western blots. The remaining mice were used for collecting metabolic data and blood flow imaging, and brains were used for immunofluorescence. In total, 23 mice were excluded due to premature death or the presence of other major health exclusions (hydrocephaly, large fighting wounds, tumors). Final group sizes ranged from 13 to 25 per group for metabolic measures, 8 to 13 for behavioral testing, and 4 to 6 for immunohistochemistry (IHC). During tests, experimenters were blinded to surgical group. Blinding to diet and sex was not possible due to mouse appearance. During analysis, experimenters were blinded to sex, diet, and dementia group.

### Surgical model of MxD

To model MxD, 3xTg-AD mice underwent a right common carotid artery occlusion surgery, as previously described, to elicit chronic cerebral hypoperfusion and model VCID [19, 31, 32]. Briefly, under isoflurane anesthesia, the right common carotid artery was ligated with two 6–0 silk sutures and cauterized (MxD group). The sham surgery (WT and AD groups only) consisted of exposing the carotid artery without ligation. Incision sites were closed with Vetbond, and the mice were given 100µL 0.03 mg/mL buprenorphine via subcutaneous injection twice per day for 3 days as an analgesic.

### Glucose tolerance test

As previously described [19, 29, 33], mice were given a glucose tolerance test (GTT) to assess diabetic status at ~ 5.75 months of age. The mice were fasted overnight, and their fasting blood glucose levels were measured (t = 0) using a glucometer (Verio IQ, OneTouch, Sunnyvale CA, USA) from their tail vein. Following an i.p. injection of 2 g/kg of glucose, blood glucose levels were measured at 15, 30, 60, 90, and 120 min post-injection to assess glucose tolerance.

### Behavior testing

Following a 2-week recovery post-GTT, mice were tested for exploratory activity and anxiety-like behavior in the open field (day 1), episodic-like memory in the novel object recognition test (NORT; day 2), spatial learning and memory in the Morris water maze (MWM; days 8–10 or 10–12), and activities of daily living using a nest building task (days 15–16). Videos were recorded of behavioral performance for open field, NORT, and MWM and analyzed using automated tracking software (ANY-maze 5.1, Stoelting, Wood Dale, IL). For each test, mice were placed into the procedure room under dim light and allowed to acclimate for 1 h. Each test apparatus was cleaned with 70% ethanol between each mouse to remove olfactory cues.

### Open field

The mice were placed in the test apparatus (495 × 495 mm box) for 10 min. Distance traveled was used to determine the general activity levels of the animal. The percent of time spent in center of the arena was used to determine anxiety-like behavior. One mouse was excluded from this test for being a statistical outlier via Grubb’s outlier test leaving group sizes of 8–13/group.

#### NORT

NORT consisted of two, five-minute trials performed in the same open field arena. In the first trial, mice were placed in the box and allowed to explore two identical objects (rubber ducks). Mice were then returned to a recovery cage for 1 h. For the second trial, mice were returned to the arena, with the one familiar object replaced with a novel object (saltshaker). Episodic-like memory was assessed by recognition index [(time with novel object/total time with objects) *100]. Between tests, objects were cleaned with 70% ethanol to mask olfactory cues. Mice that spent less than ≤ 2 s with the objects were excluded (total of 26 mice: 0–4/group, 12 males and 14 females). Additionally, 2 statistical outliers were removed from this test (1 WT LF M and 1 WT HF M).

#### MWM

Hippocampus-dependent spatial learning and memory were assessed using a modified 3-day version of the MWM that has been shown to be optimal for older, cognitively impaired, obese mice [34]. The protocol has been previously described in detail [19]. On day 1, 5 visible trials were performed in which mice learn to find the platform with a visual cue (flag). The entry point was alternated for each trial. On day 2, mice underwent 5 hidden trials, in which the visual cue was removed from the platform. All trials were 3 min long with a 30-min intertrial interval. The distance traveled to reach the platform (pathlength) was used as a measurement of non-spatial (visual trials) and spatial (hidden trials) learning. On day 3, a single probe trial was performed in which the platform was removed from the pool. Spatial memory was calculated as the percent of time spent in the target quadrant of the pool during the first minute of the probe trial. A total of 2–4 mice were excluded from this test for being statistical outliers via Grubb’s outlier test [4 mice in the hidden trial and 2 mice in the probe trial] leaving group sizes of 8–13/group.

#### Nest building

Mice were singly housed in Allentown cages with pine chip bedding and two pre-weighed Nestlets each. After 16 h (overnight), the mice were removed from their test cage and returned to group housing. Nests were rated on a 1–5 scale (with half-point scores allowed) based on published criteria [35] by 3 experimenters who were blinded to treatment group. The 3 ratings were averaged. One mouse was excluded from this test for being a statistical outlier via Grubb’s outlier test leaving group sizes of 8–13/group.

### Cerebral blood flow measurement

Cerebral blood flow was measured via laser speckle contrast imaging (moor FLPI full field laser perfusion imager; Moor Instruments, Wilmington, DE, USA) under isoflurane anesthesia as previously described [19]. Image acquisition [5-min scan], processing, and analysis were performed using moorFLPI Review V4.0 software (Moor Instruments, Wilmington, DE, USA). Average flux values were extracted from regions of interest (ROIs) using a published protocol [36]. Measurements are presented as %difference in blood flow between the left (non-ischemic for MxD mice) and right (ischemic for MxD mice) hemisphere.

### Immunofluorescence

Mice were perfused with ice-cold 0.9% saline. Brains were removed and fixed in 4% paraformaldehyde for 24 h, followed by immersion in 30% sucrose for at least 72 h. Brains were then snap frozen in OCT and stored at − 80 °C until sectioning. Brains were sectioned at 40 microns on a Leica CM1950 cryostat into 6 series. Sections were washed in PBS containing 0.01% sodium azide, permeabilized at room temperature for 1 h (0.3% TPBS), and blocked for 1 h at room temperature in 4% donkey serum in 0.3% TPBS before being incubated in blocking buffer with primary antibodies at 4 °C overnight. Primary antibodies for one series included rabbit anti-beta Amyloid (1:300, Cat# 71-5800, Lot# SH257822; Invitrogen, Waltham, MA). Primary antibodies for another series included rat anti-glial fibrillary acidic protein (1:2500, AB5804, Millipore, Lot # TA265137) or goat anti-Iba1 (1:1000, PA5-18,039, Lot #TI2638761, SJ2467805; ThermoFisher, Waltham, MA). Sections were incubated with secondary antibodies and DAPI (1:1000, Cat# D1306, ThermoFisher, Waltham, MA) in blocking buffer for 2 h at room temperature. Secondary antibodies used included Rhodamine Red-X Donkey Anti-Rabbit (1:100), Alexa Fluor 647 Donkey Anti-Goat (1:300), DyLight™ 405 AffiniPure Donkey Anti-Rat (1:300) (Jackson ImmunoResearch, West Grove, PA), Alexa Fluor® 647 AffiniPure Donkey Anti-Goat IgG (H + L) (1:1000, Cat# AB_2340437, Jackson ImmunoResearch, West Grove, PA), and Alexa Fluor® 488 Donkey Anti-Rabbit (1:1000, Cat# ab150073, Jackson ImmunoResearch, West Grove, PA). Images for quantification were taken at 10 × using the Axio Observer fluorescent microscope (Carl Zeiss Microscopy, Jena, Germany). All analyses were performed in the right hemisphere of coronal sections unless otherwise noted by an experimenter who was blinded to treatment group. For Aβ plaque quantification, 8 40-micron-thick sections between − 1.46 and − 3.52 mm from bregma were used to hand count numbers of Aβ plaques in the left and right hemisphere (Fig. 4B). For intracellular Aβ quantification, regions of interest (ROI) were drawn in the cortex and a number of cells positive for Aβ were counted (Additional file 5: Fig. S5A). For neuroinflammation analysis (microglia and astrocytes), ROIs were drawn in CA1, CA2, CA3, and dentate gyrus of the hippocampus in ~ 3 brain sections per mouse and % area covered was measured using ImageJ.

### Western blot

Frozen hippocampi (ipsilateral to VCID or sham surgery) from 4 mice per group were thawed and homogenized in 50µL RNA-Later (45-R0901-100MLsigma). 25µL of that homogenate was transferred into 100 µL T-Per buffer (ThermoScientific #78,510) supplemented with protease and phosphatase inhibitor cocktail (HALT, ThermoScientific #1861284) and spun at 21,000 g for 20 min. The supernatant was collected, and the protein concentration was determined using a Pierce BCA Protein Assay Kit (Thermo Scientific #23,227). 20 µg of protein was denatured in LDS (4× Bolt™ LDS Sample Buffer, Invitrogen, B0007) with 0.2 M DTT and boiled for 5 min at 95 °C. Proteins were then separated using a 10% Tris Bis gel (ThermoFisher, NW00107BOX) before being transferred onto a nitrocellulose membrane (Nitrocellulose/Filter Paper Sandwich, 0.2 μm, 8.3 × 7.3 cm, ThermoFisher, LC2000). Membranes were blocked using LI-COR blocking solution (Intercept® (TBS) Blocking Buffer, LI-COR, 927–60,001) for one hour at room temperature then exposed to primary antibodies at 4 °C overnight. The following day membranes were washed then incubated with the corresponding secondaries before being washed and scanned using an Odyssey CLx LI-COR scanner. All membranes were scanned simultaneously to ensure similar exposure and scanning parameters. Membranes were then stripped using LI-COR stripping buffer (NewBlot™ IR Stripping Buffers for NIR Western Blots, LI-COR, 928–40,028) for 20 min and rescanned to ensure loss of signal before being incubated with an anti-GAPDH antibody (Sigma, 45-G9545-100UL) as a loading control. Western blot band analysis was conducted using the LI-COR Image Studio and normalized to loading control. Antibodies used were for total tau ((T46) mouse abCam203179, lot #GR33658561), early phosphorylated tau (S199-202, rabbit Invitrogen 44-768G lot SG255287), and later phosphorylated tau (PHF1, rabbit Invitrogen PA5-56,621 lot TB2525148). All primary antibodies were used at 1:1000 in Tris-buffered saline supplemented with 0.1% Tween (TBST). Secondary antibodies were used at 1:15,000 in TBST with 0.02% SDS. Secondary antibodies included IRDye® 800CW Donkey anti-Mouse IgG (CAT# 926–32212, Lot#D00930-09, LI-COR, Lincoln, Nebraska) and IRDye® 680RD Donkey anti-Rabbit IgG (CAT# 926–68,073, Lot#D00421-09, LI-COR, Lincoln, Nebraska).

### Enzyme-linked immunoassay (ELISA)

The pellet from the protein extraction (insoluble fraction) was dissolved in 50ul of freshly prepared 70% formic acid and stored at -80C. On the day of the assay, the samples (n = 4 mice/group) were thawed and homogenized then spun at 4C 12,100 g for 30 min. The supernatant (40uL) was retrieved and neutralized with 20 volumes of neutralizing solution (800uL; 1 M Tris base, 0.5 M Na2HPO4, 0.05% NaN3). A protease and phosphatase cocktail was added (HALT, ThermoScientific #1861284) that contains the serine protease inhibitor ABESF to prevent degradation of AB peptides. Amyloid peptides were then quantified in this fraction using ELISA kit from ThermoFisher Scientific (AB40: KHB3481, and AB42: KHB3441) according to the manufacturer’s instructions. Quantities were normalized to total soluble protein quantities.

### Quantitative reverse transcriptase-PCR (RT-qPCR)

Frozen hippocampi (ipsilateral to VCID or sham surgery) from 4 to 5 mice per group were thawed and homogenized in 50µL RNA-Later (45-R0901-100MLsigma). RNA was extracted from 25uL of homogenate using the RNeasy® Plus Mini Kit (Qiagen, Catalog number 74134) and RNA concentrations were measured using ThermoScientific NanoDrop One. RNA was converted to cDNA using a High-Capacity cDNA Reverse Transcription Kit (Applied Biosystems, Catalog number: 4368814). The qPCR reactions were performed using TaqMan Gene Expression Master Mix (Applied Biosystems, Catalog number 4369016) in the presence of TaqMan Assays with primer/probes for Iba1 (Mm00479862_g1), GFAP (Mm01253033_m1), and CD68 (Mm00839636_g1) as target genes. RPL13A (Mm05910660_g1) was used as the housekeeping gene. Data were presented as fold change relative normalized expression compared to WT LF mice by ΔΔCq method using Bio-Rad CFX Maestro software.

### Statistics

Statistical analyses were performed using Prism 8.1 (GraphPad Software, San Diego, CA, USA). All data are presented as mean + SEM, with significance set at p < 0.05 except for categorical data (nest building is expressed as median + interquartile range). Following a Grubbs’ test for statistical outliers, a 2-way ANOVA was performed with Tukey’s correction for multiple comparisons [dementia type (WT vs. AD vs. MxD) X diet (LF vs. HF)] in data segregated by sex. The exception to this is the test for spatial learning (Morris water maze hidden trial, average pathlength) in which we used Dunnett’s multiple comparison test. We used this post hoc test because we specifically defined the deficit in average pathlength as greater than the LF WT group. In secondary analyses to assess sex differences, 3-way ANOVAs were performed (sex X dementia type X diet) without post hoc analysis (except for analysis in which WT groups were not included we used Sidak’s multiple comparison test). In measures with large sample sizes (n ≥ 13 for all groups) a ROUT test was performed (metabolic data only). One-sample t-tests were performed for measurements where values are compared to chance (50% in NORT, 25% in MWM probe trial, and 0% hemispheric difference in blood flow). Each correlation matrix was created by computing Pearson correlation coefficients for each pair of datasets.

## Results

### Animal models and timeline

In order to create a mouse model of MxD, 3xTg-AD mice (~ 3 months of age) underwent a unilateral common carotid artery occlusion surgery to induce chronic cerebral hypoperfusion/vascular pathology. These mice were compared to AD mice (3xTg-AD that underwent a sham surgery) and WT controls (WT mice that underwent a sham surgery). In order to determine the effects of HF diet-induced prediabetes on outcomes in the AD and MxD models, one week following surgery, mice were placed on either a HF diet (60% fat) or a control LF diet (10% fat) from ~ 3 to 7 months of age. A study timeline is shown in Fig. 1A.

### HF diet caused greater metabolic impairment in AD and MxD females compared to males.

We have previously reported [29] that metabolic effects of a HF diet are more severe in 3xTg-AD females compared to males. Here we show that these sex differences persist in the MxD model. Within each sex, there was a main effect of HF diet to increase % weight gain (*p* < 0.0001; Fig. 1B), visceral fat accumulation (*p* < 0.0001; Fig. 1C), and glucose intolerance (*p* < 0.0001; Fig. 1D, E). Monthly weigh gain is shown in Additional file : Figure S1A. Importantly, as others have reported in previous studies [27, 28], the HF diet induced a prediabetic phenotype with significantly elevated fasting blood glucose levels (Fig. 1D, zero time point and Additional file 1: Figure S1B) that are below the 250 mg/dl cut-off that has been established in the literature for mouse models of diabetes [37]. Of note, the highest fasting blood glucose among any of our HF-fed mice was still only 221 mg/dl. Dementia X diet interactions were sex dependent. In females, post hoc tests showed that HF-fed AD and HF-fed MxD females had greater metabolic impairment (weight gain, visceral fat accumulation, and glucose intolerance) than HF-fed WT females (*p* < 0.0001 for all measures). Conversely, in males, LF-fed AD and LF-fed MxD males had less visceral fat than LF-fed WT males (*p* < 0.001 for AD, *p* < 0.05 for MxD). Analysis of sex differences via a 3-way ANOVA (Additional file 6: Table S1) showed a sex X dementia interaction and sex X diet interaction (*p* < 0.0001 for each interaction) in which both visceral fat and glucose intolerance were exacerbated in AD/MxD and HF-fed females, but not males. Further, there was also a sex X diet X dementia interaction for % weight gain (p < 0.0001), which was driven by the large % weight increase in HF-fed AD and HF-fed MxD females. Taken together, the data show that HF diet caused greater metabolic impairment in AD or MxD females compared to WT females or AD or MxD males.

### HF diet caused a wider array of cognitive deficits in females

**Fig. 2:**
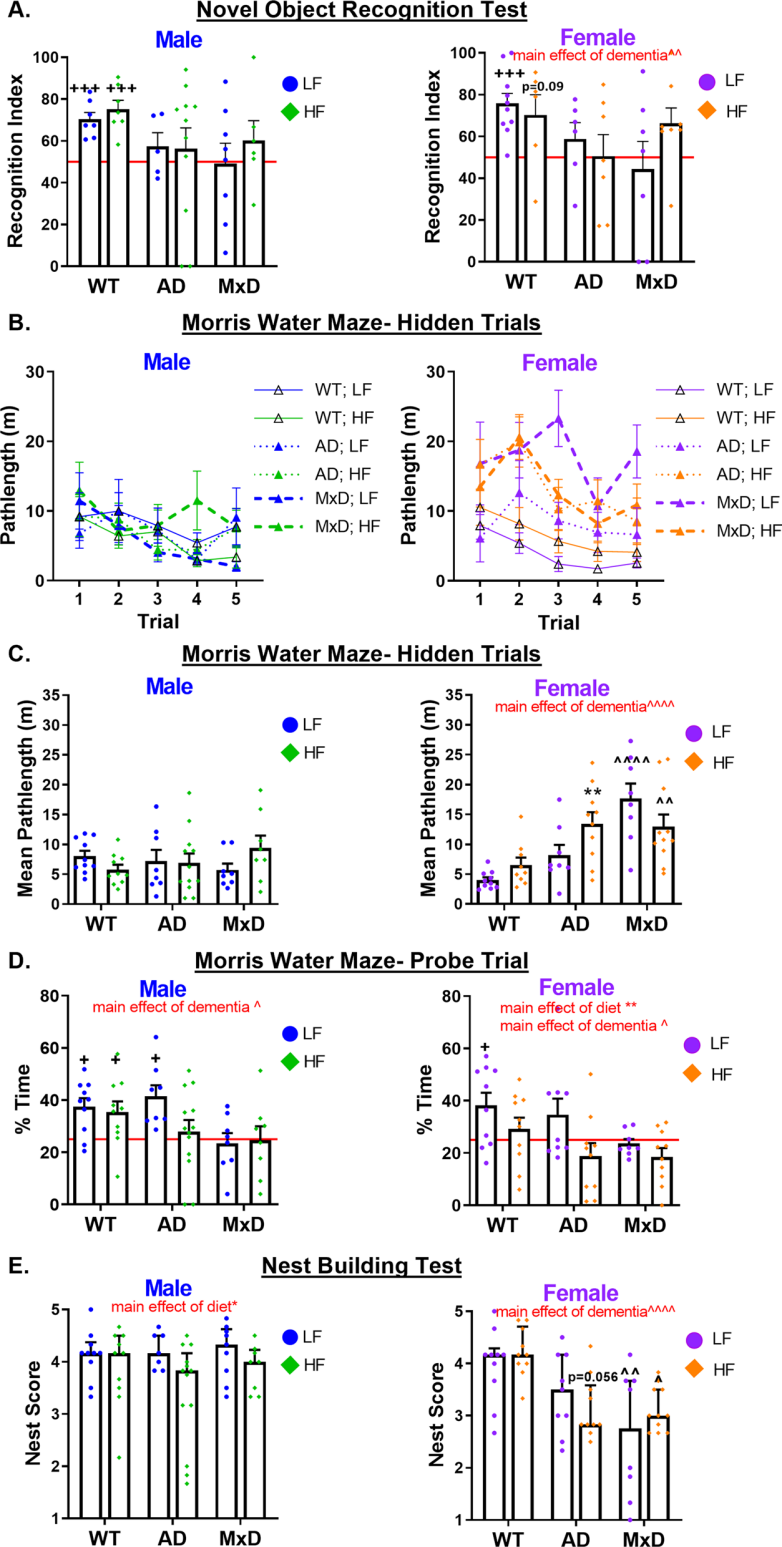
HF diet caused a wider array of cognitive impairment in females compared to males. **A** Episodic-like memory was assessed in the novel object recognition test (NORT). Recognition index (% time spent with the novel object) was calculated. Performance not significantly greater than chance (50%, indicated by the red line) indicates impaired memory. **B**–**D** Spatial learning and memory was assessed using the Morris Water Maze (MWM). Five hidden trials (**B**) assessed spatial learning via pathlength to reach the target platform (shorter pathlength = better performance). Average pathlength over the 5 hidden trials (**C**) was longer (more impaired memory) in MxD females and AD females on a HF diet. Spatial memory was assessed in the probe trial (**D**), as % time spent in the target quadrant vs. chance (25%, indicated by the red line). Performance above 25% indicates intact memory. **E** The nest building task was used to assess activities of daily living. Nests were graded on 1–5 scale (average of scores by 3 experimenters blinded to treatment). Lower scores are indicative of impairment. Data are presented as mean + SEM except for nest building (median + interquartile range), +++*p* < 0.001 vs chance, +*p* < 0.05 vs. chance, **p < 0.01 effect of diet, ^*p* < 0.05 effect of dementia, ^^*p* < 0.01 effect of dementia, ^^^^*p* < 0.0001 effect of dementia, Red line = chance, 2-way ANOVA, (*n* = 5–11/group NOR, *n* = 8–13/group MWM, nest building)

Our prior work has demonstrated that HF diet causes a wider array of cognitive deficits in middle-aged females, compared to males, in a mouse model of VCID [19]. Whether HF diet would differentially impact AD and MxD in each sex was unknown. Preference for the novel object in the NORT (episodic-like memory; Fig. 2A) was assessed with a one-sample t-test vs. no preference (recognition index of 50%). In males, all WT mice demonstrated a preference for the novel object (LF-fed WT p < 0.001, HF-fed WT p < 0.001). In females, LF-fed WT mice show intact memory (*p* < 0.001), while HF-fed WT females only showed a trend toward preference for the novel object (*p* = 0.09). In both sexes, both AD and MxD groups on either diet demonstrated no preference for the novel object, indicating an impairment in episodic-like memory. A 3-way ANOVA showed no sex differences. Although there were some group differences in exploratory behavior and anxiety-like behavior in the open field (Additional file 2: Figure S2A, B), neither of these measures correlated with NORT performance (Additional file 1: Figure S1C). There were no significant differences in performance during the NORT training trial (Additional file 1: Figure S1D). The MWM was used to examine spatial learning and memory. All groups were trained to swim to a visible platform (main effect of trial *p* < 0.05, Additional file 2: Figure S2E). When the platform was hidden (spatial learning trials; Fig. 2B, C), no group differences were observed in males. In females, there was a dementia x diet interaction (*p* < 0.05), in which HF-fed AD females, LF-fed MxD females, and HF-fed MxD females all had significantly impaired spatial memory compared to LF-fed WT females (*p* < 0.01 for each group; post hoc tests). A 3-way ANOVA (Additional file 6: Table S1) showed a sex X dementia X diet interaction (*p* < 0.01) in which HF-fed AD/MxD females had the most severe spatial learning deficits. In the probe trial (spatial memory; Fig. 2D), preference for the target quadrant was assessed with a one-sample t-test of % time in target quadrant vs. chance (25%). In males, only WT mice (on either diet) and LF-fed AD mice showed a preference for the target quadrant (p < 0.05), indicating that spatial memory was impaired in HF-fed AD males and MxD males on either diet. In females, only the LF-fed WT mice showed a preference for the target quadrant (*p* < 0.05), indicating that HF-fed WT females and AD or MxD females on either diet had impaired spatial memory. A 3-way ANOVA (Additional file 6: Table S1) showed only a slight trend toward worse spatial memory in females (*p* = 0.08) with no significant interactions. Due to known decreases in swim speed in obese mice, we used non-speed-based measures (pathlength/% time in the target quadrant). Although there were some group differences in swim speed, speed did not correlate with learning or memory in the MWM (Additional file 2: Figure S2F), further supporting that cognitive measures were speed independent. The nest building test (Fig. 2E) was used to assess activities of daily living (ADLs). In males, all mice performed well although there was a main effect of diet (*p* < 0.05) to impair ADLs. In females, AD and MxD females showed clear impairments (main effect of dementia *p* < 0.0001), with LF-fed MxD (*p* < 0.01) and HF-fed MxD (*p* < 0.05) females building poorer nests than control LF-fed WT females. HF-fed AD females also showed a trend (*p* = 0.056) toward impaired ADLs. A 3-way ANOVA showed a main effect of sex X dementia interaction (*p* < 0.01) in which AD and MxD females were more impaired than males. Taken together, these data show a wider array of cognitive deficits in AD or MxD females compared to males, particularly when females were fed a HF diet.

### Neuropathology was exacerbated in AD/MxD females.

**Fig. 3:**
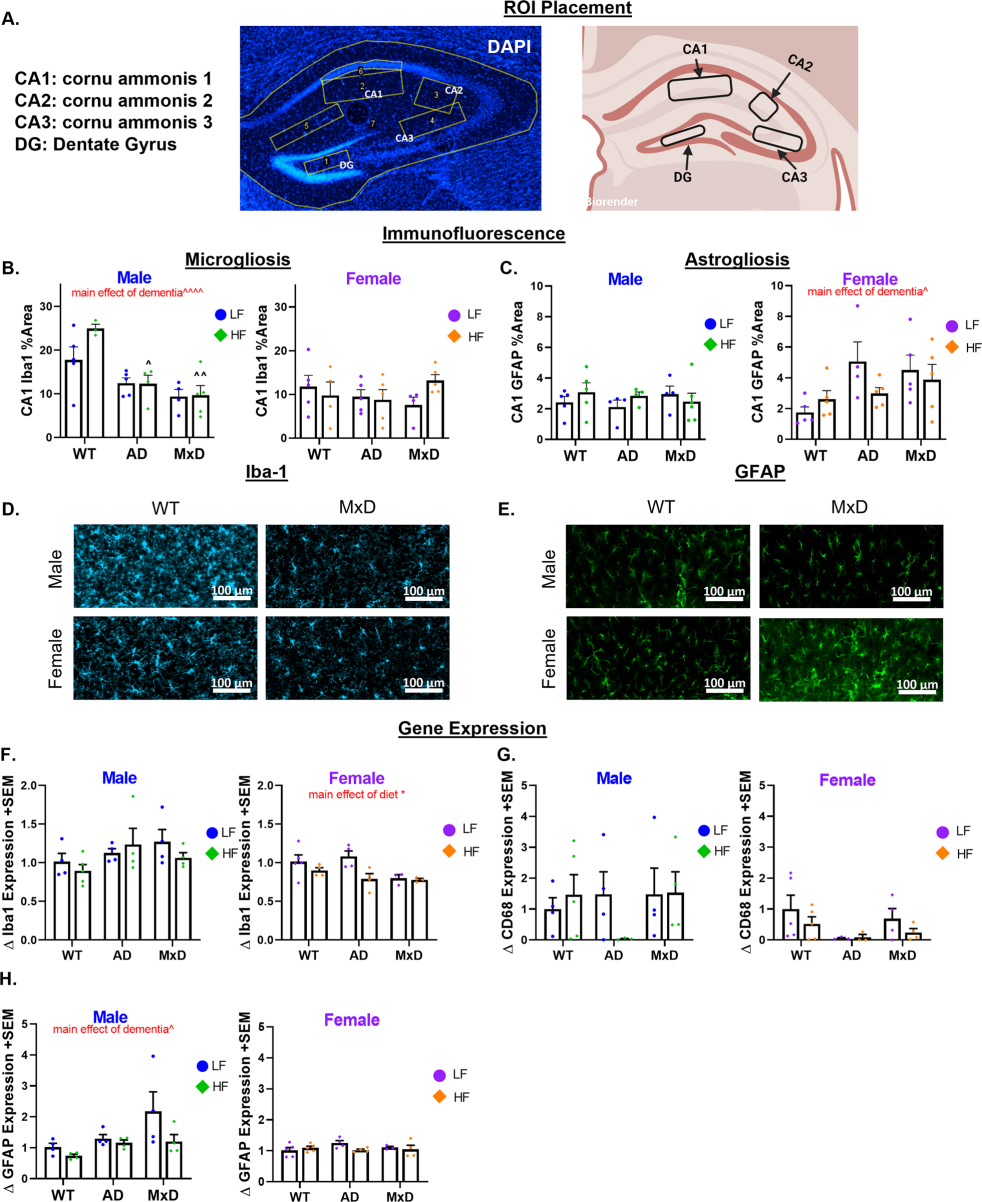
Astrogliosis is exacerbated in AD/MxD females, while microglia coverage is decreased in AD/MxD males. **A** Hippocampal regions of interest examined: CA1, CA2, CA3, and the dentate gyrus (image created with BioRender.com). **B** Microgliosis in the CA1 region of the hippocampus was gauged through Iba1 immunofluorescence (greater % area covered indicating greater microgliosis). **C** Astrogliosis was gauged through GFAP immunofluorescence (greater % area covered indicating greater astrogliosis). **D** Representative images of Iba-1 immunofluorescence in the CA1 regions. **E** Representative images of GFAP immunofluorescence in the CA1 regions. **F**–**H** Hippocampal expression of markers for microgliosis (Iba-1: F and CD68:** G**) and astrogliosis (GFAP: **H**) normalized to RPL13A expression. Data are presented as mean + SEM, effect of dementia: ^*p* < 0.05 effect of dementia, ^^^^*p* < 0.0001 effect of dementia, effect, *p < 0.05 effect of diet, 2-way ANOVA, (*n* = 4–5/group)

**Fig. 4:**
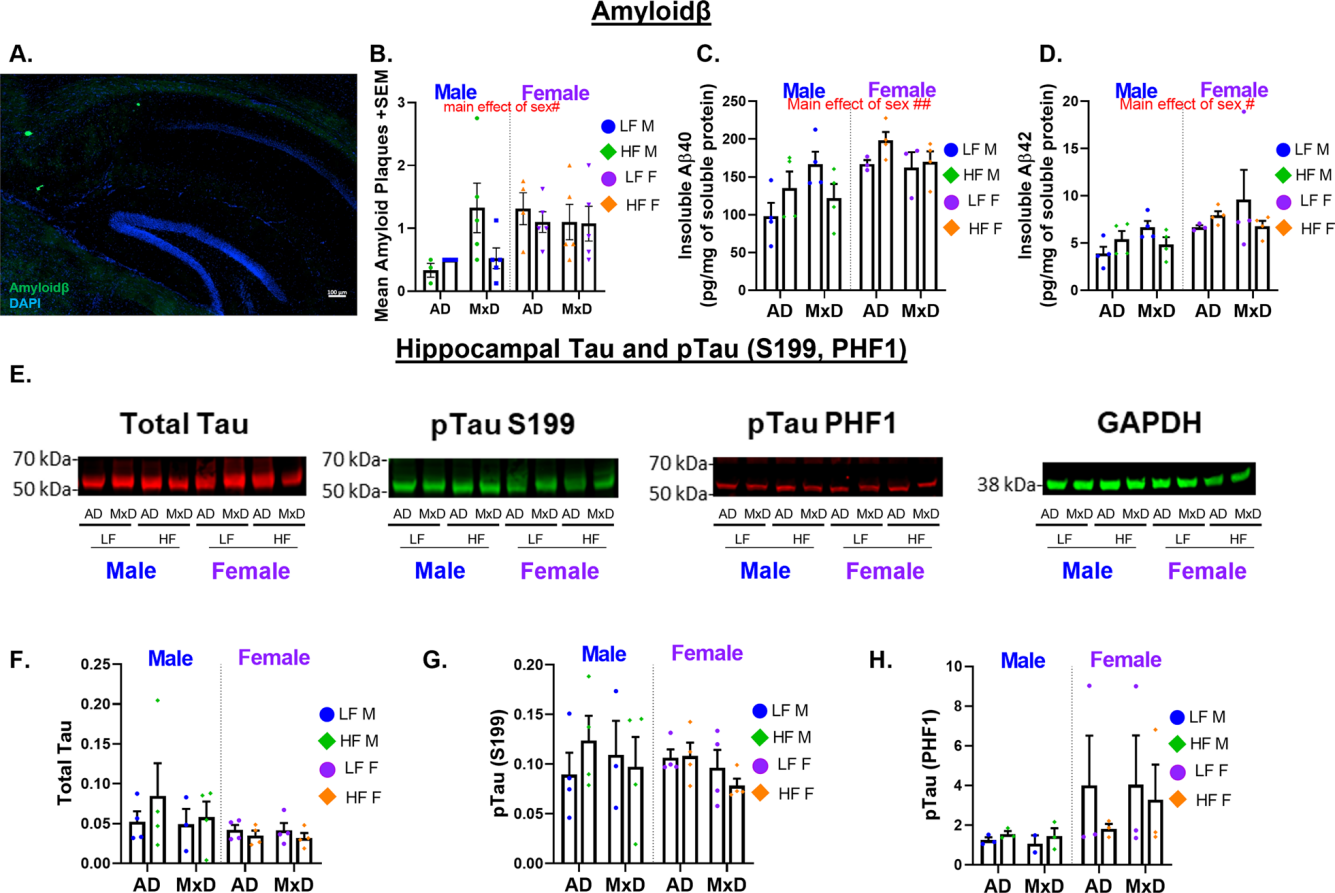
Female AD/MxD mice have greater Aβ, but not tau, pathology. **A** Representative image of Aβ plaques. **B** The number of Aβ plaques/brain section (averaged across 8, 40-μM-thick brain sections between -1.46 and -3.52 mm from bregma). **C** Hippocampal insoluble Aβ-40, measured by ELISA. **D** Hippocampal insoluble Aβ-42, measured by ELISA. **E** Representative Western blot images of tau protein in right (ischemic for MxD) hippocampal isolates. Measurements for **F** total tau, **G** pTau S199, and **H** pTauPHF1 were normalized to GAPDH. Data are presented as mean + SEM, #*p* < 0.05 effect of sex, ##*p* < 0.01 effect of sex, 3-way ANOVA, (*n* = 4–5/group)

We have previously reported sex-dependent effects of HF diet on hypothalamic neuroinflammation in 3xTg-AD mice; however, neuroinflammation in brain areas associated with cognition and other forms of neuropathology had not been assessed. Microgliosis (immunolabeling for Iba-1) and astrogliosis (immunolabeling for glial fibrillary acid protein; GFAP) were assessed in the hippocampal CA1, CA2, CA3, and dentate gyrus regions (Fig. 3A). In males, there was a main effect of dementia on microgliosis (Iba1% area covered; Fig. 3B, D, Additional file 3: Figure S3A–C, and Additional file 4: Figure S4A), in which AD and MxD males showed decreased hippocampal microgliosis in several regions (CA1 *p* < 0.001, CA2 *p* < 0.0001, CA3 *p* < 0.01). In females, microgliosis was unaffected by either diet or dementia in any of the regions assessed. A 3-way ANOVA showed a sex X dementia interaction in several regions (CA1 *p* < 0.01, CA2 *p* < 0.01, CA3 *p* < 0.05), which was driven by higher microgliosis in WT males (regardless of diet) compared to all other groups. In males, astrogliosis (GFAP % area covered; Fig. 3C, E, Additional file 3: Figure S3D-F, and Additional file 4: Figure S4B) was unaffected by either diet or dementia in any of the regions assessed. In females, there was a main effect of dementia, in which AD and MxD females showed increased hippocampal astrogliosis (CA1 *p* < 0.05, CA2 *p* < 0.01, dentate gyrus *p* < 0.01). A 3-way ANOVA showed a sex X dementia interaction in which AD/MxD females had greater astrogliosis than males (CA1 *p* < 0.05, CA2 *p* < 0.05, CA3 *p* < 0.01, dentate *p* < 0.05). In addition to examining % area covered, we assessed expression of hippocampal genes associated with microgliosis (Iba-1 and CD68, a lysosomal protein upregulated during microglial activation: Fig. 3F, G) and astrogliosis (GFAP: Fig. 3H). In males, GFAP gene expression in hippocampal homogenate was increased overall by dementia (*p* < 0.05). However, GFAP gene expression was unaffected in females. CD68 expression was unaffected by diet or dementia in both males and females. In females, HF diet decreased Iba-1 expression (*p* < 0.05). To examine AD pathology, we assessed Aβ pathology via immunohistochemistry for Aβ, ELISA for insoluble Aβ40 and 42 in the hippocampus, and Western blot for phosphorylated tau in the hippocampus. Soluble Aβ was not assessed, as it is not elevated until 18 months of age in 3xTg-AD mice [38]. A 3-way ANOVA showed that females had higher levels of Aβ plaques than males (*p* < 0.05; Fig. 4A, B) and higher levels of insoluble Aβ40 (*p* < 0.01; Fig. 4C) and 42 (*p* < 0.05; Fig. 4D) compared to males. Cortical intracellular Aβ was also assessed, in which a 3-way ANOVA showed that AD/MxD females had higher levels of Aβ-positive cells than males (p < 0.0001) and that there was a main effect of MxD to increase Aβ (*p* < 0.01; Additional file 5: Figure S5A). No differences in phosphorylated or total tau were detected between AD and MxD groups, regardless of sex or diet (Fig. 4E-H). To validate that the unilateral carotid artery occlusion surgery modeled VCID by inducing chronic cerebral hypoperfusion, cortical blood flow was measured using laser speckle contrast imaging at ~ 7 months of age (4 months post-surgery). As expected, blood flow deficits were found in the right (occluded) hemisphere of all MxD groups (main effect of MxD, *p* < 0.0001; Additional file 5: Figure S5B, C). No sex differences were detected. Taken together, these data show that although some pathology was equivalent between the sexes, hippocampal astrogliosis and Aβ pathology were more severe in AD/MxD females, compared to males.

### Sex-dependent correlations between cognitive performance and metabolic measures

**Fig. 5:**
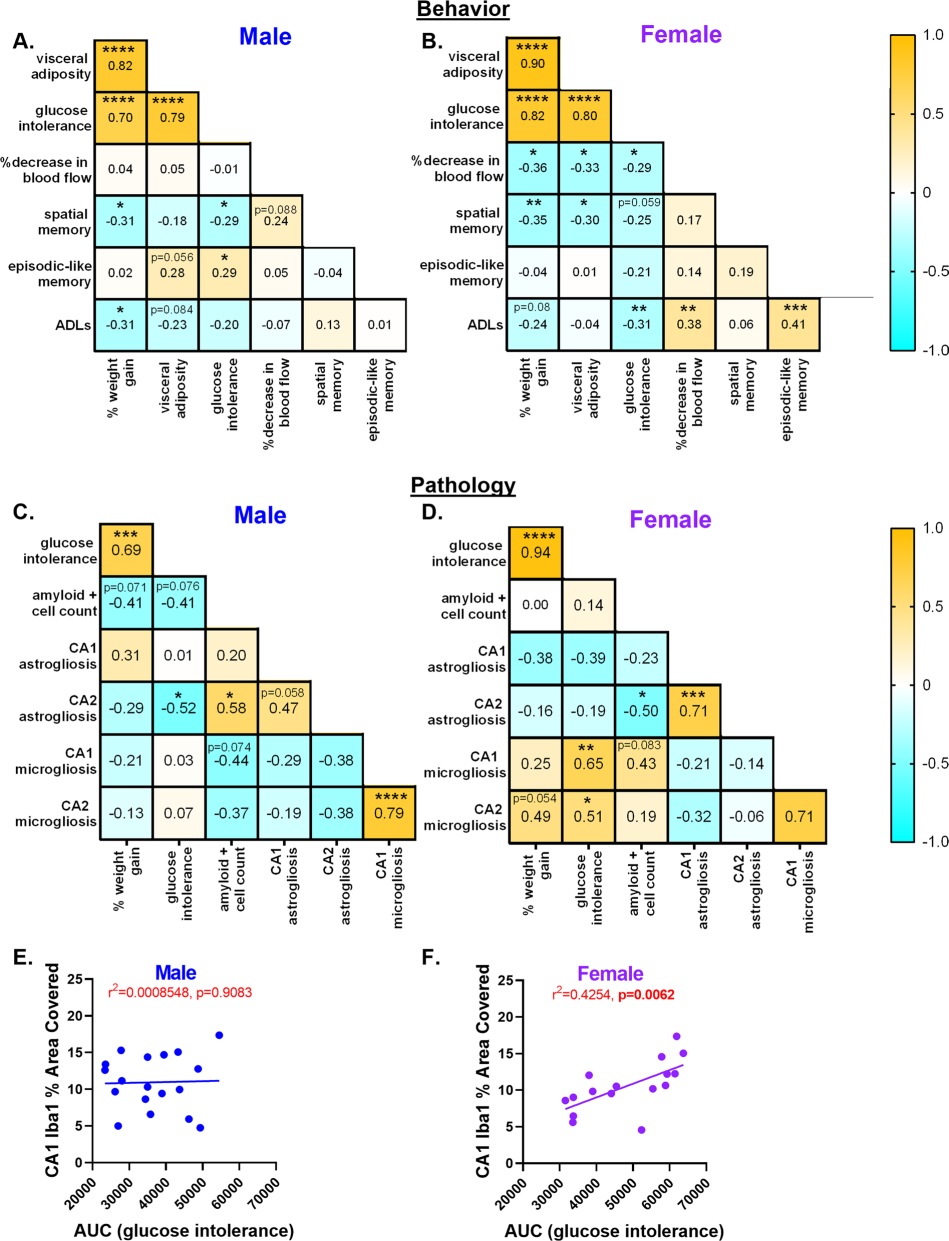
Cognitive impairments and pathological outcomes are correlated with metabolic deficits in male and female mice. Using a correlation matrix, we compared relationships between cognitive, metabolic, and pathological factors. **A**, **B** Correlations between cognitive and metabolic measures. Visc fat: visceral fat pad weight normalized to body weight (*n* = 55–58/sex); AUC: area under the curve from the glucose tolerance test, high AUC indicates greater glucose intolerance (*n* = 54–58/sex); %decrease in blood flow: the percent difference in cerebral blood flow in the temporal region of the cortical brain surface (*n* = 50–51/sex); MWM % target: % of the time spent in the target quadrant of the probe trial of the MWM test, higher percentage indicates better spatial memory (*n* = 58/sex); NOR% time: % time spent with the novel object in the testing trial of the NOR test, higher percentage indicates better episodic-like memory (*n* = 46–49/sex); ADLs: activities of daily living assessed by score in the nest building test, a lower score indicates worse ADLs (*n* = 57–58/sex). **C**, **D** Correlations between metabolic and pathological measures in the subset of mice designated for IHC. AUC: area under the curve from the glucose tolerance test, high AUC indicates greater glucose intolerance (*n* = 18–21/sex); amyloid count: the number of cells in a cortical ROI that were positive for beta-amyloid (*n* = 19–20/sex); CA1 GFAP: the % area covered by GFAP staining in the CA1 region of the hippocampus, greater coverage indicates greater astrogliosis in that region (*n* = 18–19/sex); CA2 GFAP, (*n* = 17–19/sex); CA1 Iba2: the % area covered by Iba1 staining in the CA1 region of the hippocampus, greater coverage indicates greater astrogliosis (*n* = 17–18/sex) CA2 Iba1, (*n* = 18/sex). *p < 0.05, **p < 0.01, ***p < 0.001, ****p < 0.0001, significant correlation; Pearson r values are presented. Yellow: positive correlation, Blue: negative correlation. **E**, **F** Linear regression of CA1 Iba1% area covered and glucose tolerance test from the AUC (males: *n* = 19; females *n* = 17). *r*^2^ value and *p* value are presented

To further examine the relationship between cognitive, metabolic, and neuropathology measures, we performed linear regression analyses and correlation analyses in males and females separately. In Fig. 5A, B, we examined correlations between behavioral outputs and measurements of metabolic impairment in males and females separately. In both males and females, each metabolic measure was strongly correlated with the others (visceral fat, % weight gain, and AUC; *r* = 0.7–0.9, *p* < 0.0001). In males, the effects of metabolic impairments on cognitive function were mixed. In males, higher % weight gain and more severe glucose intolerance (GTT AUC) were both associated with poorer spatial memory (MWM % time in the target quadrant; *p* < 0.05 for each). Additionally in males, % weight gain was associated with reduced ability to perform activities of daily living (nest score; *p* < 0.05); however, more severe glucose intolerance showed a surprising association with better episodic-like memory (NORT recognition index; *p* < 0.05). In females, metabolic impairment showed a consistent association with worse cognitive function. In females, higher % weight gain or more visceral fat was associated with poorer spatial memory (*p* < 0.01 for weight gain, *p* < 0.05 for visceral fat), and more severe glucose intolerance was associated with reduced ability to perform activities of daily living (*p* < 0.01). In males, the degree of metabolic impairment was not associated with alterations in cerebral blood flow, and blood flow was not associated with cognitive function. In females, metabolic impairment was associated with reduced cerebral blood flow (% blood flow in the right hemisphere; *p* < 0.05 for % weight gain, visceral fat, and glucose intolerance). Additionally in females, greater reduction in cerebral blood flow was associated with more severe deficits in the ability to perform activities of daily living (nest building). Taken together, these data show that impairment in metabolic measures was more consistently associated with reductions in cognitive function in females.

In Fig. 5C, D we examined correlations between neuropathological and metabolic outputs and presented our findings in the form of a correlation matrix. In males, greater cortical Aβ burden was associated with more CA2 astrogliosis (*p* < 0.05), while in females it was associated with less CA2 astrogliosis. In males, more severe glucose intolerance was associated with less CA2 astrogliosis (*p* < 0.02), and there were no significant associations with microgliosis. In females, more severe glucose intolerance was associated with more microgliosis in both CA1 (*p* < 0.01) and CA2 (*p* < 0.05), but not associated with astrogliosis. An example of a sex-specific association is shown in Fig. 5E, F in which we examined the relationship between microgliosis in the CA1 region (CA1 Iba1% area covered) and glucose intolerance (AUC) separately in males and females via linear regression and presented the r^2^ and* p* value. Taken together, these data show that more severe glucose intolerance was the only metabolic parameter that was associated with worse neuroinflammation, and that this increase in neuroinflammation (microgliosis) occurred in females only.

**Fig. 6:**
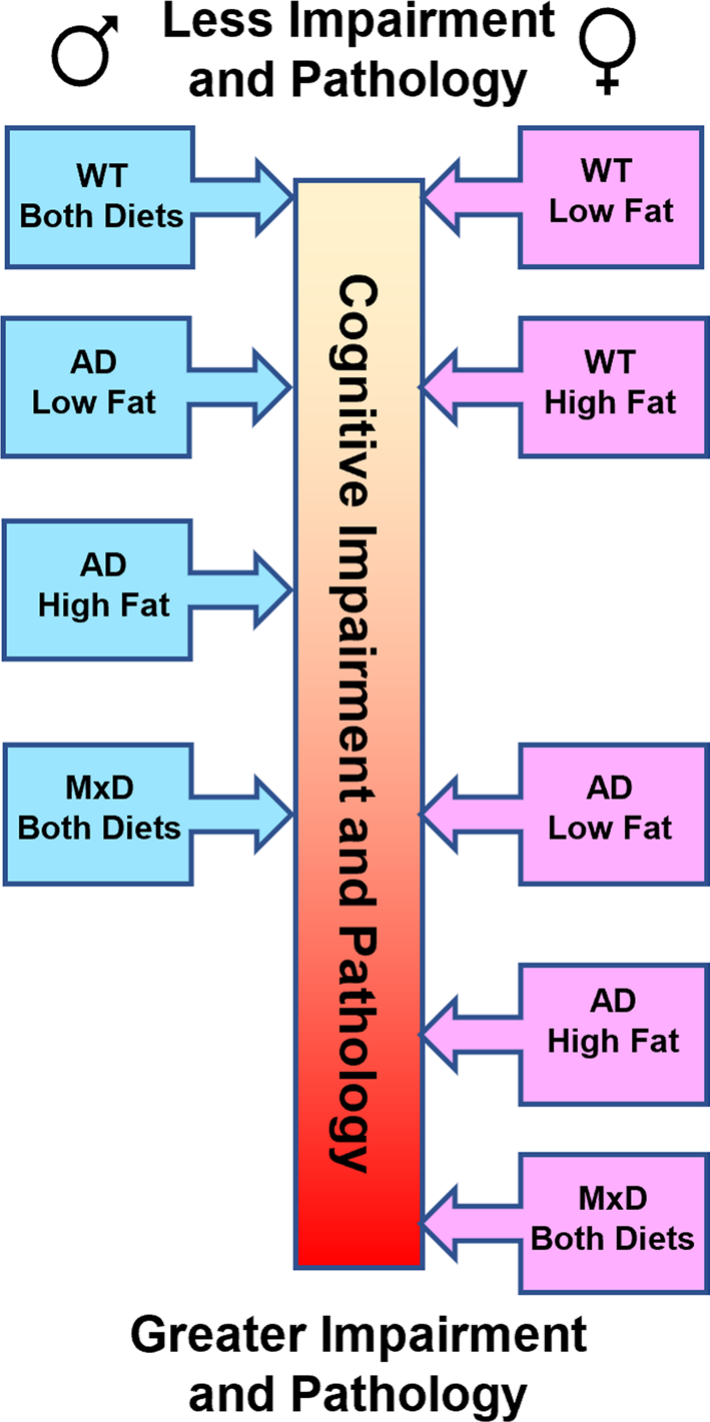
Summary of our major findings. We demonstrate relative accumulation of cognitive impairments and neuropathology of female male (left, blue) and (right, pink) mice along a center scale with the top of the scale indicating less impairment and pathology and the bottom of the scale representing greater impairment and pathology

A summary of findings is presented in Fig. 6.

## Discussion

This study set out to address gaps in knowledge regarding sex differences in mid-life metabolic risk factors for dementia – obesity and prediabetes. Recent clinical evidence suggests that prediabetes is a risk factor for cognitive decline and dementia in women, but not men [17]. How sex and prediabetes interact to influence cognitive function and neuropathology in the two most common forms of dementia – AD and MxD (co-morbid VCID + AD pathology) – is unknown. To fill this gap in knowledge, we combined mouse models of VCID (chronic cerebral hypoperfusion) and AD (3xTg-AD mice) to model MxD. We used a chronic HF diet to model obesity and prediabetes. While HF diet ubiquitously caused metabolic impairments, this was augmented in AD/MxD females, but not males. AD, MxD, and HF diet differentially affected cognition in males and females. While episodic-like memory deficits were observed in AD and MxD mice of both sexes, additional cognitive impairments were observed in females. AD/MxD females also showed impairments in activities of daily living, but males did not. When fed a HF diet, AD and MxD females, but not males, also exhibited deficits in spatial learning. Metabolic impairment was also more consistently associated with reductions in cognitive function in females. Moreover, AD and MxD females had more hippocampal astrogliosis and a greater burden of Aβ than males. Finally, more severe glucose intolerance was associated with worse microgliosis in females only. Taken together, the data demonstrate heightened susceptibility of females to the negative metabolic, cognitive, and neuropathological effects of AD, MxD, and HF diet. These data, along with recent clinical findings [17], suggest that prediabetes might contribute to multiple forms of dementia in women.

### Metabolic findings

Here, we found that AD and MxD females had more adverse metabolic responses to chronic HF diet. We have previously shown that sex differences in response to HF diet are age sensitive. If the diet is initiated in juvenile mice, males show greater metabolic impairments [33]. This sex difference is eliminated if the diet is initiated in adulthood [33], and reversed if initiated in middle age – with females showing greater metabolic impairment [19, 33]. In line with our prior findings, in the current study, the diet was initiated in young adulthood and thus WT males and females showed similar levels of metabolic impairment. However, AD/MxD females developed greater metabolic impairments, including increased weight gain and more severe glucose intolerance, than males in response to HF diet. In a subset of mice from the current study (WT and AD groups with the sham surgery), we previously reported that 3xTg-AD females did not display increased plasma levels of other diabetes markers (e.g., insulin, GIP, GLP-1, PAI-1, resistin) or peripheral inflammation [29]. This enhanced metabolic effect observed in AD/MxD females in the current study is reminiscent of our findings in middle-aged mice in which females are more adversely affected than males. One possibility is that the AD and MxD females might have displayed a form of accelerated aging of their metabolic system and/or brain regulation of metabolic function. We have previously reported this female-specific interaction between the effects of a HF diet and the 3xTg-AD genotype that worsens metabolic impairment and examined potential underlying mechanisms [29]. In a subset of mice from the current study (WT and AD groups with the sham surgery), we reported increased food intake, reduced activity levels, and markedly increased hypothalamic expression of GFAP and IL-1β, as well as GFAP labeling in several hypothalamic nuclei that regulate energy balance in the HF-fed 3xTg-AD females [29]. Thus, neuroinflammation in the hypothalamus likely contributes to the observed sex difference in metabolic function in 3xTg-AD mice. A limitation in our interpretation of these data is in the generalizability of using a diet composed of 60% fat from lard (saturated fat). Diets of different fat composition or combinations with sugar (a Western diet) may have different consequences. While we were unable to address changes in blood pressure in this study, others have demonstrated that high-fat diet in wild-type mice and in the 5xFAD mouse model of AD does not increase blood pressure [39]. Overall, our results show that a chronic 60% fat diet caused greater metabolic impairment in AD/MxD females compared to males.

### Cognitive findings

Although HF diet elicited some cognitive deficits in both sexes, HF-fed females displayed a wider array of cognitive impairments, even in WT mice (summarized in Fig. 6). While numerous studies have shown that HF diet exacerbates cognitive impairment in AD [20–23, 25], few have examined sex differences. Here, we report that HF diet caused sex-specific cognitive deficits in spatial learning. AD males, regardless of diet, did not show impairment in spatial learning; however, AD females showed an impairment in spatial learning only when fed a HF diet. In both males and females, additive effects of HF diet were observed in spatial memory. In males, LF-fed AD mice did not show spatial memory deficits; however, with a combination of HF diet and AD these impairments emerged. In females, even WT mice showed spatial memory impairment in response to HF diet (as did AD mice). Overall, we found that poorer metabolic measures were also more consistently associated with poorer cognitive function in females, compared to males. In addition to the sex differences observed with HF diet, we also observed a wider array of cognitive impairment in LF-fed AD females compared to LF-fed males. In support of the current data, prior studies have demonstrated exacerbation of cognitive deficits in 3xTg-AD mice by a HF diet [20–25] and greater cognitive impairments in AD females compared to males [40–45]. However, there have been some reports of greater deficits in males specifically in regard to working memory [46] and fear conditioning [47]. Greater female susceptibility to negative cognitive effects of a HF diet is in line with our prior work demonstrating that HF diet also causes a wider range of cognitive deficits in WT middle-aged females [19]. Taken together, our data suggest that females may be more adversely cognitively affected by metabolic disease which could contribute to cognitive deficits during normal aging or dementia.

In both sexes, MxD elicited a wider array of cognitive deficits beyond what was observed in the AD model, with females being most impacted. Among LF-fed mice, spatial learning deficits emerged in MxD females that were not observed in the AD females or MxD males. Among LF-fed males, spatial memory deficits emerged with MxD that were not observed in AD males but were observed in AD/MxD females. Due to floor effects, we were unable to detect effects of MxD in females for some tests or to detect the effect of HF diet in the MxD groups in either sex for most tests. However, it is important to note that MxD females were impaired in all four cognitive tests, while MxD males had preserved spatial learning and ability to perform activities of daily living. Our MxD model involved a unilateral common carotid artery occlusion surgery, which leads to chronic cerebral hypoperfusion [31–49] and cognitive deficits even in WT mice [19, 31, 50, 32]. While others have examined the pathological effects of chronic cerebral hypoperfusion in 3xTg-AD mice [51], we are the first to examine sex differences in this model. Our data support that added vascular pathology in MxD further exacerbates cognitive deficits, compared to AD alone, and that females may be more adversely cognitively affected by chronic cerebral hypoperfusion in MxD.

### Neuropathology findings

In assessing underlying neuropathology, we found that astrogliosis and Aβ accumulation were more severe in AD/MxD females, compared to males. First, we assessed markers of neuroinflammation. Female AD/MxD mice had greater astrogliosis than males in several regions of the hippocampus. We, and others, have previously reported increased astrogliosis in 3xTg-AD mice [29, 40] and in cerebral hypoperfusion mouse models of VCID [32, 52, 53]. In comparison to our 7-month-old mice, greater microglia activation has been reported in 12-month-old 3xTg-AD females compared to males [54], and more Iba1 + cells in the subiculum in 18-month-old 3xTg-AD females compared to males [55]; thus, sex differences may have emerged at later time points. Although there were no sex differences in the extent of microgliosis, we did observe sex differences in the relationship between microgliosis and metabolic markers. In females, but not males, more severe glucose intolerance was associated with more severe microgliosis in the CA1 region of the hippocampus; mechanistic links driving this outcome require further investigation. In addition to neuroinflammation, we assessed other classic AD neuropathology, including Aβ and phosphorylated tau. While we observed no group differences in hippocampal phosphorylated tau levels, recent work by the LaFerla lab has demonstrated sex differences in hippocampal tau in the 3xTg-AD mouse[55]. Overall, we found that females had greater levels of amyloid pathology both in terms of Aβ plaque counts as well as in Aβ 40 and 42 levels in the hippocampus with no added effects of diet or MxD. Others have found increased Aβ levels in female 3xTg-AD mice [21, 40, 54–58] as well as increased Aβ-positive cell counts in the dorsal hippocampus [44]. Others have found no hypoperfusion-induced elevation in Aβ levels in 3xTg-AD mice [51]; however, this was after only 2 months of hypoperfusion suggesting that changes we observed may have emerged at a later time point. While we did not observe an effect of diet on Aβ pathology, others have found that HF diet increases Aβ in 3xTg-AD mice [21, 24, 25, 59], with some studies showing that this effect is specific to females [57, 58]. This may be due to increased Aβ production that can occur in metabolic disease, which is exacerbated when plaques have formed [60]. While further sex differences in dementia pathology may develop with age, increased Aβ and astrogliosis in females support the heightened sensitivity of females to AD/MxD and may underlie the wider array of cognitive deficits in females.

In addition to AD pathology, we also assessed the degree of chronic cerebral hypoperfusion. We found that unilateral carotid artery occlusion surgery (MxD mice) caused deficits in cerebral blood flow in the cortical surface that persisted 4 months after surgery. This is in line with our previous work, and that of others, documenting prolonged deficits in blood flow in the ipsilateral hemisphere [19, 31–49, 61]. While we observed the expected effect of MxD to cause hypoperfusion, we did not observe any sex or diet differences. This is in line with a previous study by Bracko et al. showing that HF diet-induced obesity do not cause hypoperfusion or capillary stalling in AD[62]. However, we did observe that greater metabolic impairment was associated with more severe hypoperfusion in females, but not males. Thus, the degree of metabolic impairment may be important. Pires et al. have shown that HF diet causes cerebral vessel remodeling [63]. Blood flow recovery following unilateral carotid artery occlusion surgery could be affected by the degree of collateral blood flow, angiogenesis, and compensation following surgery. A limitation of the current study is that we only examined relative blood flow in the cortical surface; there may be differences in blood flow in deeper brain structures that we were unable to detect. We have previously assessed changes in blood flow using arterial spin labeling MRI and found that although hippocampal blood flow was reduced by the unilateral common carotid occlusion surgery, it was not further reduced by diet in this deeper brain structure [61]. Sex differences in the 3xTg-AD mouse have been noted, including a greater association between plaques and markers of hypoxia [54]. These findings suggest that there could be a stronger connection between vascular dysfunction and Aβ pathology in females. Our finding that metabolic impairment is associated with more severe hypoperfusion in females only prompts further research into the potential of metabolic disease to interfere with blood flow compensation and recovery in a sex-specific manner.

There may be additional neuropathologies not addressed in the current study that could have contributed to sex differences in cognitive function. For example, we have previously shown that HF diet impairs adult hippocampal neurogenesis in WT females, but not males [18]. This deficit occurred in the dorsal hippocampus, where neurogenesis supports spatial learning and memory. Additionally, HF diet can accelerate AD pathology in 3xTg-AD mice through several mechanisms not assessed in the current study, such as by increasing neuronal cell death, oxidative stress [23], and brain atrophy [20]. Future work is needed to find mechanistic insights behind the sex differences we and others have observed. Given the greater AD pathology in females, the interconnectedness of vascular and AD pathology, and our current findings showing that female MxD mice are more adversely affected, MxD may pose a greater cognitive threat to women.

### Potential mechanisms underlying observed sex differences

Sex hormones may influence dementia on many levels, in both men and women, and their effects must be viewed with consideration to age and disease state. Estrogens are present in both sexes but are higher in reproductive-age women. Estrogen also protects against AD risk factors (diabetes) and VCID risk factors (hypertension, obesity, diabetes, stroke) in women [15]. It is unknown how estrogen would impact AD and VCID when the pathologies overlap in MxD. Generally, estrogen can protect the brain through its vasodilatory, anti-apoptotic, antioxidant, and anti-inflammatory actions [15, 64]. Rodent studies have also shown protective effects of estrogen against Aβ [59, 65, 66]. A limitation of our work is that menopause is not taken into consideration. Most women with dementia are post-menopausal, and menopause decreases estrogen levels. Thus, we are currently assessing the effects of menopause in several mouse models of dementia. While the evidence for the protective effects of estrogen is robust, we have found that AD/MxD female mice are more strongly impacted by HF diet. The 3xTg-AD genotype may diminish the capacity of the brain to produce estrogen, given that women with AD have lower levels of brain aromatase (the enzyme that produces estrogen) [65, 67] and women with AD have lower brain estrogen levels [67]. There is evidence that estrogen’s effect may turn from protective to damaging when acting in the presence of inflammation or a disease state. For example, estrogen can increase inflammation if administered in older mice after a long period of estrogen withdrawal [68] or lose effectiveness in ameliorating AD pathology [59]. Additionally, studies examining the impact of hormone replacement therapy on dementia risk have flagged diabetic women as a population in which HRT increases dementia risk [69]. Diabetes has been shown to diminish and sometimes reverse the protective effect of estrogen in rodent models of ischemia [70, 71]. In this study, chronic HF diet-induced obesity could have created an “accelerated aging” effect in females that negated the protective influence of estrogen.

Androgens may also contribute to sex differences reported here. In men, age-related androgen decline is found as a risk factor for developing AD [72]. In male 3xTg-AD mice, gonadectomy (which reduces testosterone) increases Aβ and tau pathology but is rescued by treatment with testosterone [73, 74]. While the effects of testosterone on MxD specifically are unclear, testosterone has a complicated influence on the cerebral vasculature [64, 75] with potentially harmful effects, such as increasing vasoconstriction [76–78] and inflammation [79, 80], but decreasing inflammation in disease states [81, 82] in males. Our lab has previously reviewed the cerebrovascular actions of androgens and how that relates to metabolic and cardiovascular disease [75]. More research is needed to determine if testosterone level changes in prediabetes affect long-term risk of MxD.

### Clinical relevance

Given that there are well-documented sex differences in both AD and VCID, sex differences likely play a role in MxD. Prediabetes research is clinically important because the prediabetic population is growing, there is accelerating awareness of its connection to dementia, and, importantly, it is a potentially modifiable dementia risk factor. To incorporate this risk factor into clinical prevention and treatment of dementia, more research is needed particularly in its effects on MxD, which is inadequately represented in research. Women with diabetes have greater risk of developing cognitive impairments, and in certain types of dementia experience greater cognitive impairments than men with diabetes [3, 83, 84]. Metabolic disease in general appears to be a greater dementia risk factor for women [15, 64]. This also extends to prediabetes, which is an all-cause dementia risk factor [85, 86]. A 2021 study found that prediabetes is associated with accelerated dementia onset and declining cognitive function only in women [17]. Diabetes is more aggressively treated than prediabetes; this difference may manifest in long-term consequences. For example, a recent study found a relationship between Aβ burden and prediabetes but not diabetes [87]. The authors hypothesized that this is due to beneficial effects of diabetes treatment. It is clear that further research is needed to grasp how prediabetes fits into the overall picture of sex differences in dementia.

## Conclusion

In summary, using a mouse model of mixed (AD + VCID) dementia, AD and MxD females showed a wider array of cognitive deficits, compared to males. Astrogliosis and Aβ pathology were also more severe in AD/MxD females, compared to males. When challenged with a HF diet, AD or MxD females also had increased metabolic impairment compared to males. Metabolic impairment was also more consistently associated with reductions in cognitive function in females. More severe glucose intolerance was associated with worse microgliosis in females only. Here, we demonstrate the importance of considering how sex modulates the relationship between risk factors and dementia. This work supports the importance of prediabetes as a risk factor for multiple forms of dementia, particularly for women, and emphasizes increased cognitive and pathological sensitivity to high-fat diet. Future studies assessing the overlap of other midlife risk factors, such as menopause, will be important for the development and utilization of therapeutic strategies for dementia treatment and prevention.

## Supplementary Information

**Additional file 1: Figure S1. **HF diet increased weight gain and fasting blood glucose in males and females. A) Body weight was measured monthly starting just prior to the surgery and onset of diet (“Pre”) and ending at tissue collection (“End”), (n = 15–28/group). B) Fasting blood glucose levels were measured at the beginning of the glucose tolerance test. Data are presented as mean + SEM, ^p < 0.05 effect of dementia, **** p < 0.0001 effect of diet, 2-way ANOVA, (n = 17–26/group).

**Additional file 2: Figure S2. **HF diet decreased locomotor activity in females and increased center time in MxD males and females. A) General locomotor activity was measured by tracking the distance traveled (in meters) during the open field test. B) Anxiety-like behavior and disorientation were measured using the %time that the mice spend in the center of the testing arena during the open field test. C) Correlation matrix for open field measures (distance traveled and % time in the center of the arena) and episodic-like memory as measured by the NOR recognition index (RI) for males and females. Pearson r values and p values are presented. Yellow: positive correlation, Blue: negative correlation. (n = 45–58/sex). D) Exploration time during the training trial of the NOR test. The red line marks 2 s of exploration, which was used as the cut-off for minimum object exploration to be included in the test. E) MWM visible trial (day 1) pathlength by trial. F) Correlation matrix for average swim speed in the visible trials of the MWM (avg. speed visible) and spatial learning (hidden trial) and spatial memory (probe trial). Pearson r values and p values are presented. Yellow: positive correlation, Blue: negative correlation, (n = 6–13 /group). A-B and D-E) Data are presented as mean + SEM, *p < 0.05 effect of diet, ****p < 0.0001 effect of diet, ^p < 0.05 effect of dementia, ^^p < 0.01 effect of dementia, 2-way ANOVA.

**Additional file 3: Figure S3. **Microglia coverage is decreased in AD/MxD males, while astrogliosis is exacerbated in AD/MxD females. Microglia coverage in the CA1 region of the hippocampus was gauged through Iba1 immunofluorescence (larger % area covered indicating greater microgliosis). Iba1 immunoreactivity was used to calculated microglia coverage as the percent area covered by Iba1. Hippocampal regions of interest examined: CA2 (A), CA3 (B), and the dentate gyrus (C). Astrogliosis in multiple regions of the hippocampus was gauged through GFAP immunofluorescence (greater % area covered indicating greater astrogliosis). Hippocampal regions of interest examined:CA2 (D), CA3 (E), and the dentate gyrus (F). Data are presented as mean + SEM, ^ p < 0.05 effect of dementia, ^^p < 0.01 effect of dementia, ^^^^p < 0.0001 effect of dementia, 2-way ANOVA, (n = 4–5/group).

**Additional file 4: Figure S4. **Hippocampal Iba1 and GFAP immunofluorescence intensity. Immunofluorescence intensity of Iba1 (A) and GFAP (B) was measured. Hippocampal regions of interest examined: CA1, CA2, CA3, and the dentate gyrus. There was a main effect of dementia in Iba1 intensity in males, but there were no differences in hippocampal GFAP intensity. Data are presented as mean + SEM (effect of dementia: ^ p < 0.05 effect of dementia, ^^p < 0.01 effect of dementia, ^^^p < 0.001 effect of dementia, ^^^^p < 0.0001 effect of dementia 2-way ANOVA, (n = 4–5/group).

**Additional file 5: Figure S5. **Female AD/MxD mice have greater cortical Aβ pathology, and MxD mice exhibit deficits in blood flow regardless of sex. A) To examine amyloid burden, a number of cells positive for Aβ in a cortical region of interest were counted (n = 4–5/group). B) To validate that the unilateral carotid artery occlusion surgery modeled VCID by inducing chronic cerebral hypoperfusion, cortical blood flow was measured using laser speckle contrast imaging at ~ 7 months of age [ 4 months post-surgery]. The % difference in blood flow between the ischemic and non-ischemic hemispheres with a value closer to 0 indicating no difference in blood flow and a negative % difference indicating lower blood flow in the hemisphere ipsilateral to the occlusion (n = 8–23/group). C) Representative images for blood flow scans show cerebral blood flow for sham (top) and MxD (bottom). Data are presented as mean + SEM, effect of sex #### p < 0.0001, main effect of dementia ^^p < 0.01, ^^^^p < 0.0001; 3-way ANOVA with Sidak’s multiple comparison test.

**Additional file 6: Table S1. **Analysis of sex differences. Results (p values) of 3-way ANOVAs examining main effects of sex, diet, dementia, and interaction effects. Significant results are shown in white, values trending toward significance in light gray, and non-significant results in dark gray.

## Data Availability

The datasets used and/or analyzed during the current study are available from the corresponding author on reasonable request.

## Abbreviations

AD: Alzheimer's disease
ADL: Activities of daily living
ANOVA: Analysis of variance
AUC: Area under the curve
Aβ: Amyloid beta
BACE1: Beta-secretase 1
CA1–3: Cornu Ammonis 1–3
CD68: Cluster of differentiation 68
GFAP: Glial fibrillary acidic protein
GTT: Glucose tolerance test
HF: High fat (diet)
Hif1-alpha: Hypoxia inducible factor 1-alpha
IBA1: Ionized calcium-binding adaptor molecule 1
IHC: Immunohistochemistry
IL-1β: Interleukin-1beta
LF: Low fat (diet)
MWM: Morris water maze
MxD: Mixed dementia
NORT: Novel object recognition test
SEM: Standard error of the mean
VCID: Vascular contributions to cognitive impairment and dementia
Visc: Visceral (Fat)
WT: Wild type
