# Supplementary figures and images for: High-fat diet exacerbates cognitive decline in mouse models of Alzheimer's disease and mixed dementia in a sex-dependent manner
Source: J Neuroinflammation. 2022 May 14;19:110. doi: 10.1186/s12974-022-02466-2 (PMC9107741; doi:10.1186/s12974-022-02466-2)

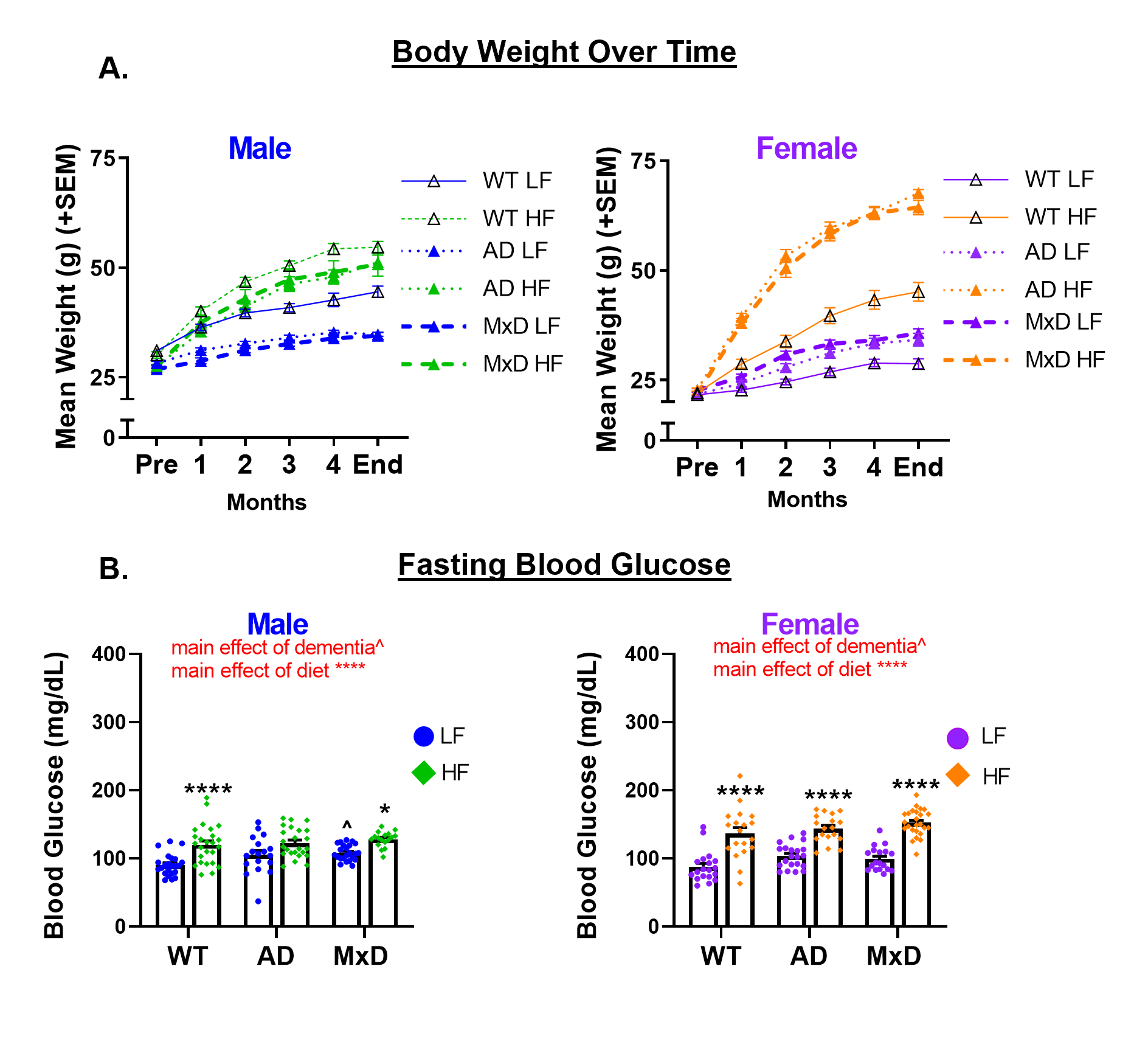

Supplement: Supplementary file 1 — Additional file 1: Figure S1. HF diet increased weight gain and fasting blood glucose in males and females. A) Body weight was measured monthly starting just prior to the surgery and onset of diet (“Pre”) and ending at tissue collection (“End”), (n = 15–28/group). B) Fasting blood glucose levels were measured at the beginning of the glucose tolerance test. Data are presented as mean + SEM, ^p < 0.05 effect of dementia, **** p < 0.0001 effect of diet, 2-way ANOVA, (n = 17–26/group). [file 12974_2022_2466_MOESM1_ESM.tif]

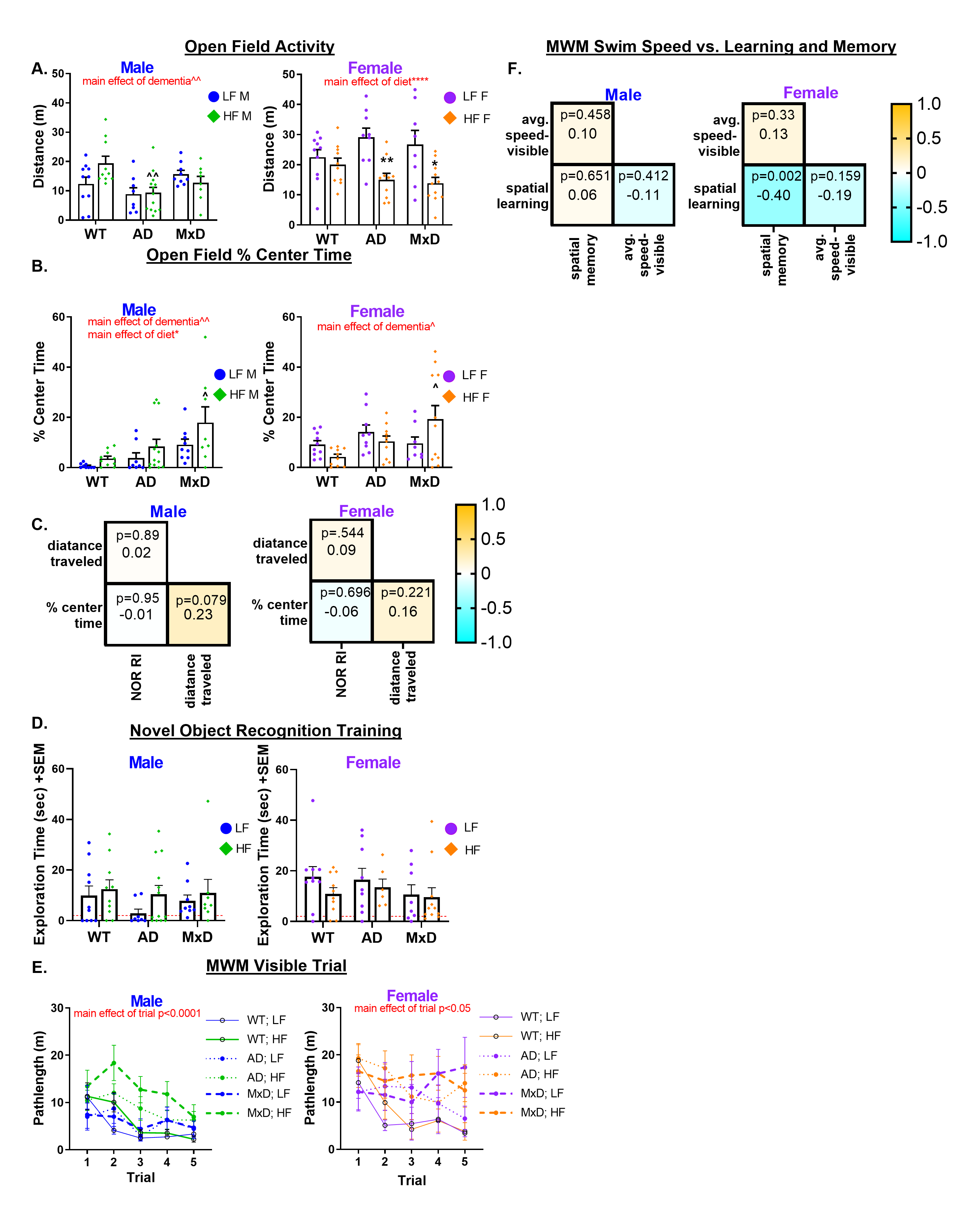

Supplement: Supplementary file 2 — Additional file 2: Figure S2. HF diet decreased locomotor activity in females and increased center time in MxD males and females. A) General locomotor activity was measured by tracking the distance traveled (in meters) during the open field test. B) Anxiety-like behavior and disorientation were measured using the %time that the mice spend in the center of the testing arena during the open field test. C) Correlation matrix for open field measures (distance traveled and % time in the center of the arena) and episodic-like memory as measured by the NOR recognition index (RI) for males and females. Pearson r values and p values are presented. Yellow: positive correlation, Blue: negative correlation. (n = 45–58/sex). D) Exploration time during the training trial of the NOR test. The red line marks 2 s of exploration, which was used as the cut-off for minimum object exploration to be included in the test. E) MWM visible trial (day 1) pathlength by trial. F) Correlation matrix for average swim speed in the visible trials of the MWM (avg. speed visible) and spatial learning (hidden trial) and spatial memory (probe trial). Pearson r values and p values are presented. Yellow: positive correlation, Blue: negative correlation, (n = 6–13 /group). A-B and D-E) Data are presented as mean + SEM, *p < 0.05 effect of diet, ****p < 0.0001 effect of diet, ^p < 0.05 effect of dementia, ^^p < 0.01 effect of dementia, 2-way ANOVA. [file 12974_2022_2466_MOESM2_ESM.tif]

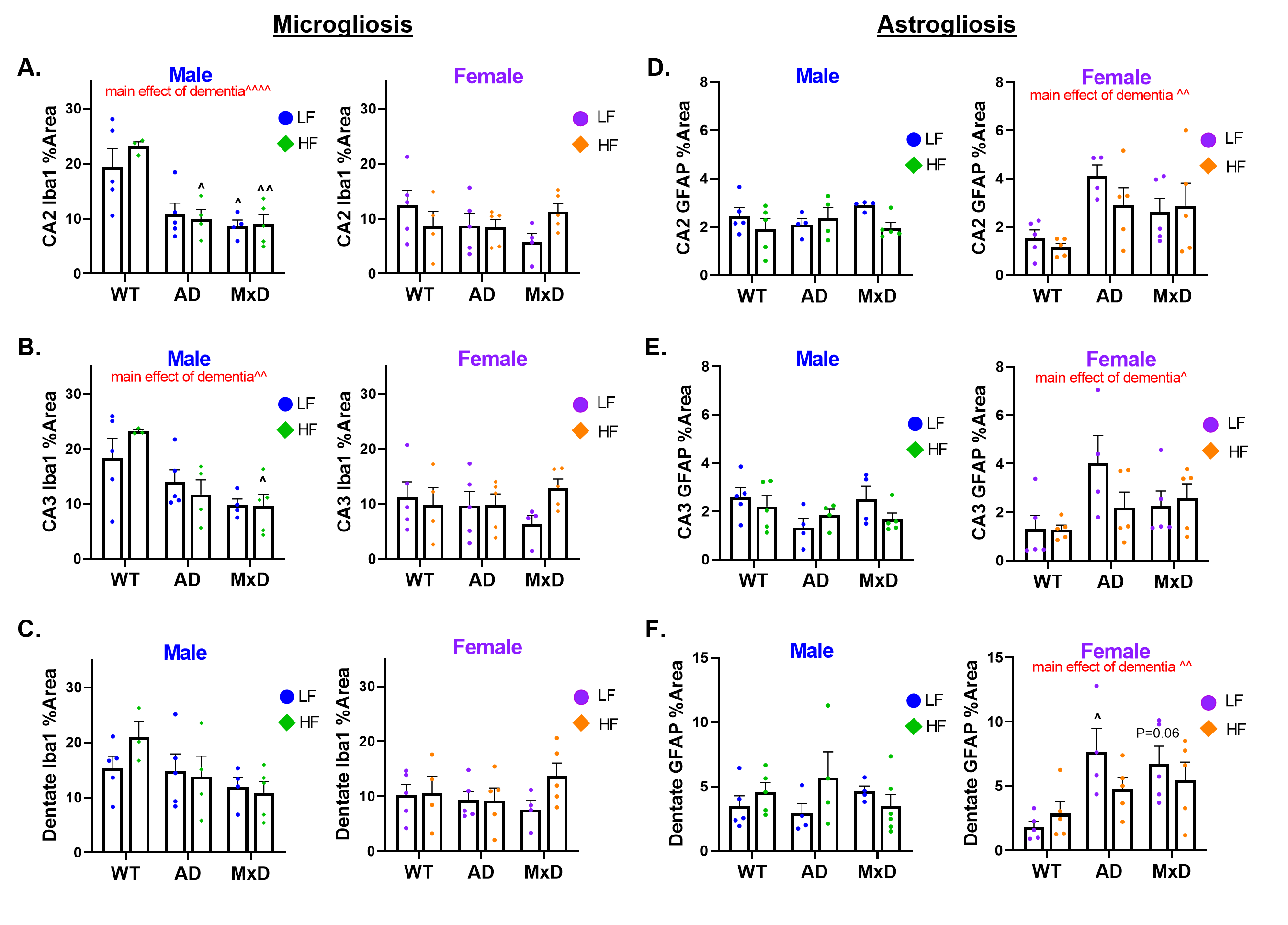

Supplement: Supplementary file 3 — Additional file 3: Figure S3. Microglia coverage is decreased in AD/MxD males, while astrogliosis is exacerbated in AD/MxD females. Microglia coverage in the CA1 region of the hippocampus was gauged through Iba1 immunofluorescence (larger % area covered indicating greater microgliosis). Iba1 immunoreactivity was used to calculated microglia coverage as the percent area covered by Iba1. Hippocampal regions of interest examined: CA2 (A), CA3 (B), and the dentate gyrus (C). Astrogliosis in multiple regions of the hippocampus was gauged through GFAP immunofluorescence (greater % area covered indicating greater astrogliosis). Hippocampal regions of interest examined:CA2 (D), CA3 (E), and the dentate gyrus (F). Data are presented as mean + SEM, ^ p < 0.05 effect of dementia, ^^p < 0.01 effect of dementia, ^^^^p < 0.0001 effect of dementia, 2-way ANOVA, (n = 4–5/group). [file 12974_2022_2466_MOESM3_ESM.tif]

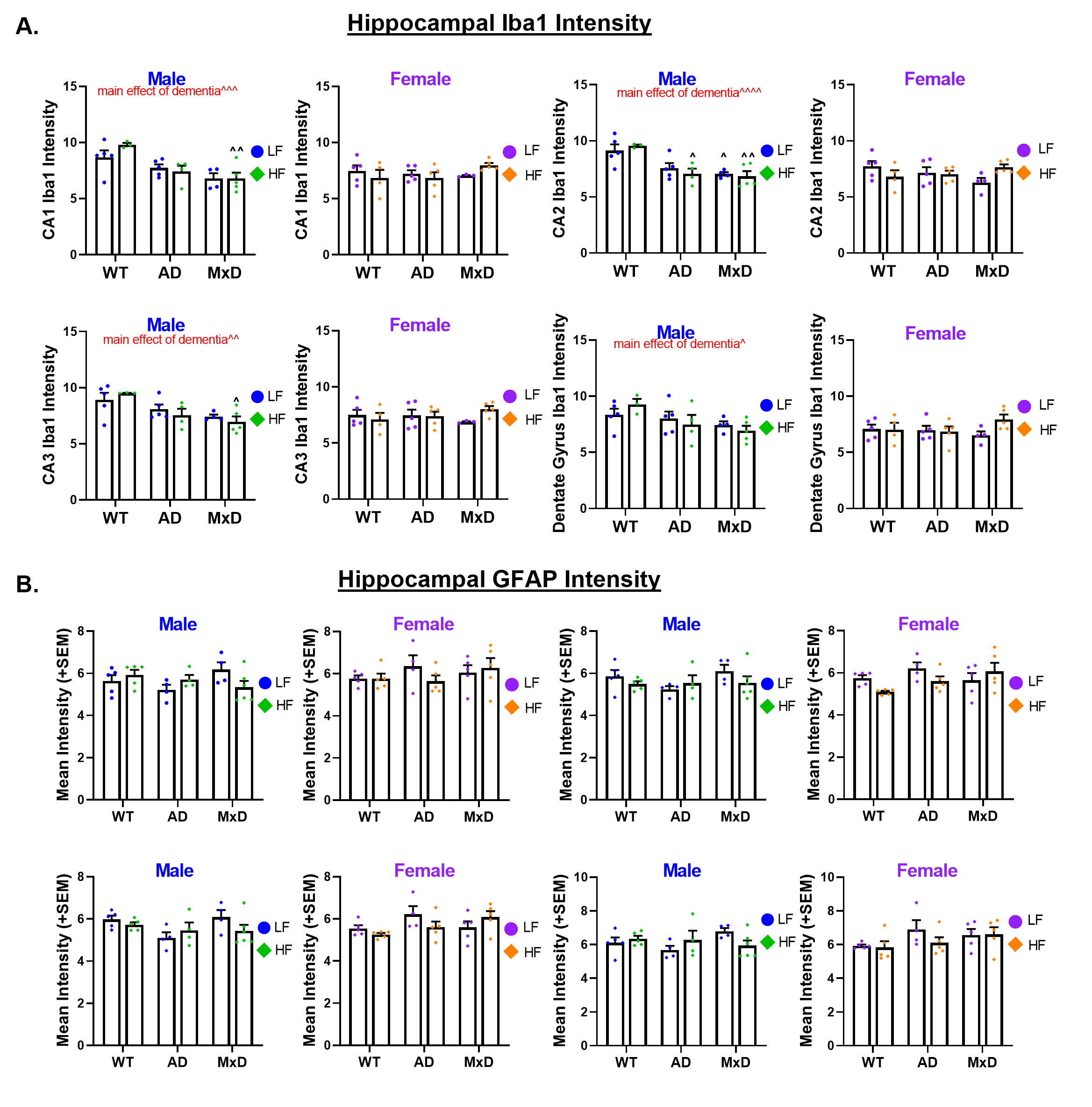

Supplement: Supplementary file 4 — Additional file 4: Figure S4. Hippocampal Iba1 and GFAP immunofluorescence intensity. Immunofluorescence intensity of Iba1 (A) and GFAP (B) was measured. Hippocampal regions of interest examined: CA1, CA2, CA3, and the dentate gyrus. There was a main effect of dementia in Iba1 intensity in males, but there were no differences in hippocampal GFAP intensity. Data are presented as mean + SEM (effect of dementia: ^ p < 0.05 effect of dementia, ^^p < 0.01 effect of dementia, ^^^p < 0.001 effect of dementia, ^^^^p < 0.0001 effect of dementia 2-way ANOVA, (n = 4–5/group). [file 12974_2022_2466_MOESM4_ESM.tif]

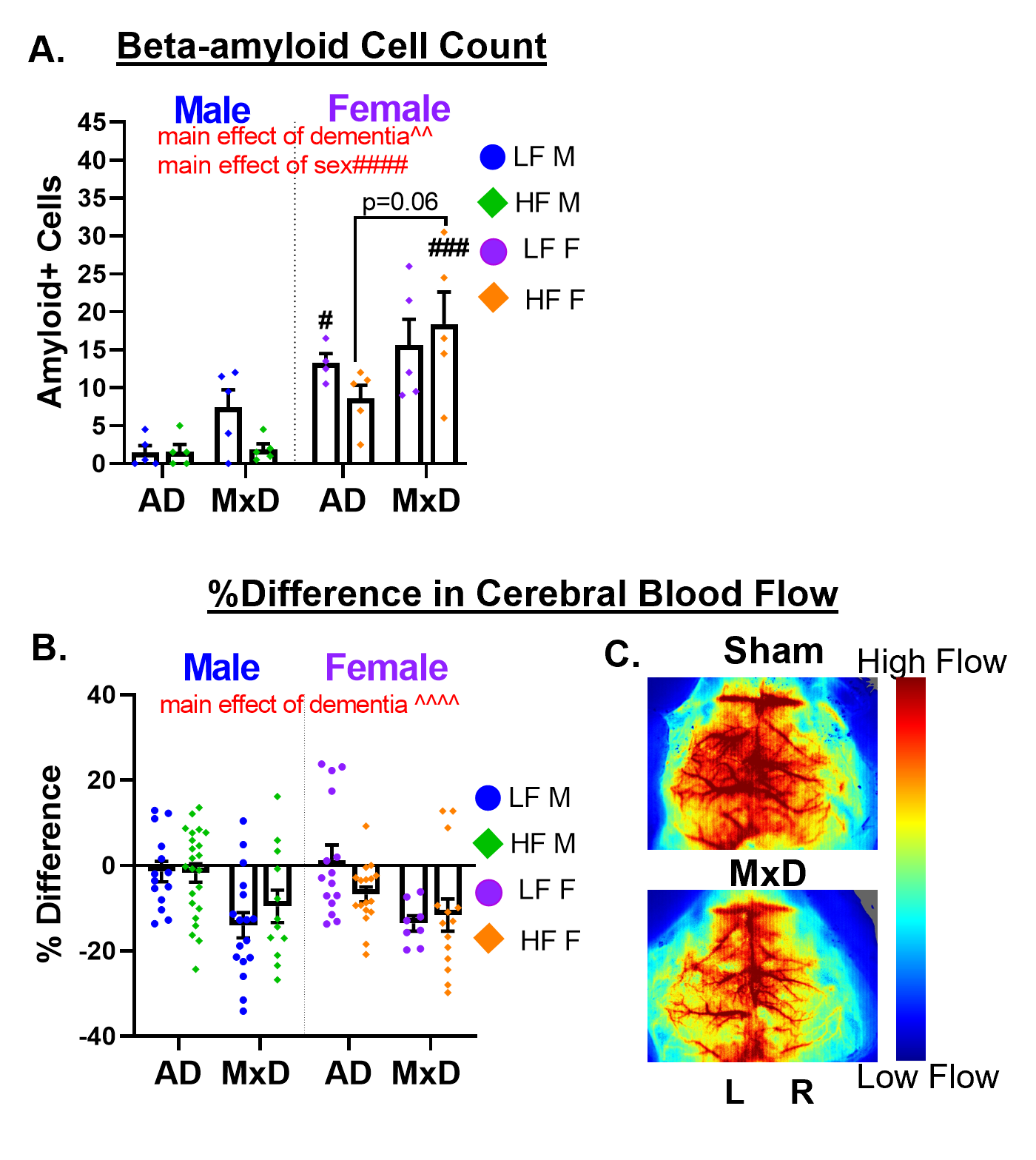

Supplement: Supplementary file 5 — Additional file 5: Figure S5. Female AD/MxD mice have greater cortical Aβ pathology, and MxD mice exhibit deficits in blood flow regardless of sex. A) To examine amyloid burden, a number of cells positive for Aβ in a cortical region of interest were counted (n = 4–5/group). B) To validate that the unilateral carotid artery occlusion surgery modeled VCID by inducing chronic cerebral hypoperfusion, cortical blood flow was measured using laser speckle contrast imaging at ~ 7 months of age [ 4 months post-surgery]. The % difference in blood flow between the ischemic and non-ischemic hemispheres with a value closer to 0 indicating no difference in blood flow and a negative % difference indicating lower blood flow in the hemisphere ipsilateral to the occlusion (n = 8–23/group). C) Representative images for blood flow scans show cerebral blood flow for sham (top) and MxD (bottom). Data are presented as mean + SEM, effect of sex #### p < 0.0001, main effect of dementia ^^p < 0.01, ^^^^p < 0.0001; 3-way ANOVA with Sidak’s multiple comparison test. [file 12974_2022_2466_MOESM5_ESM.tif]

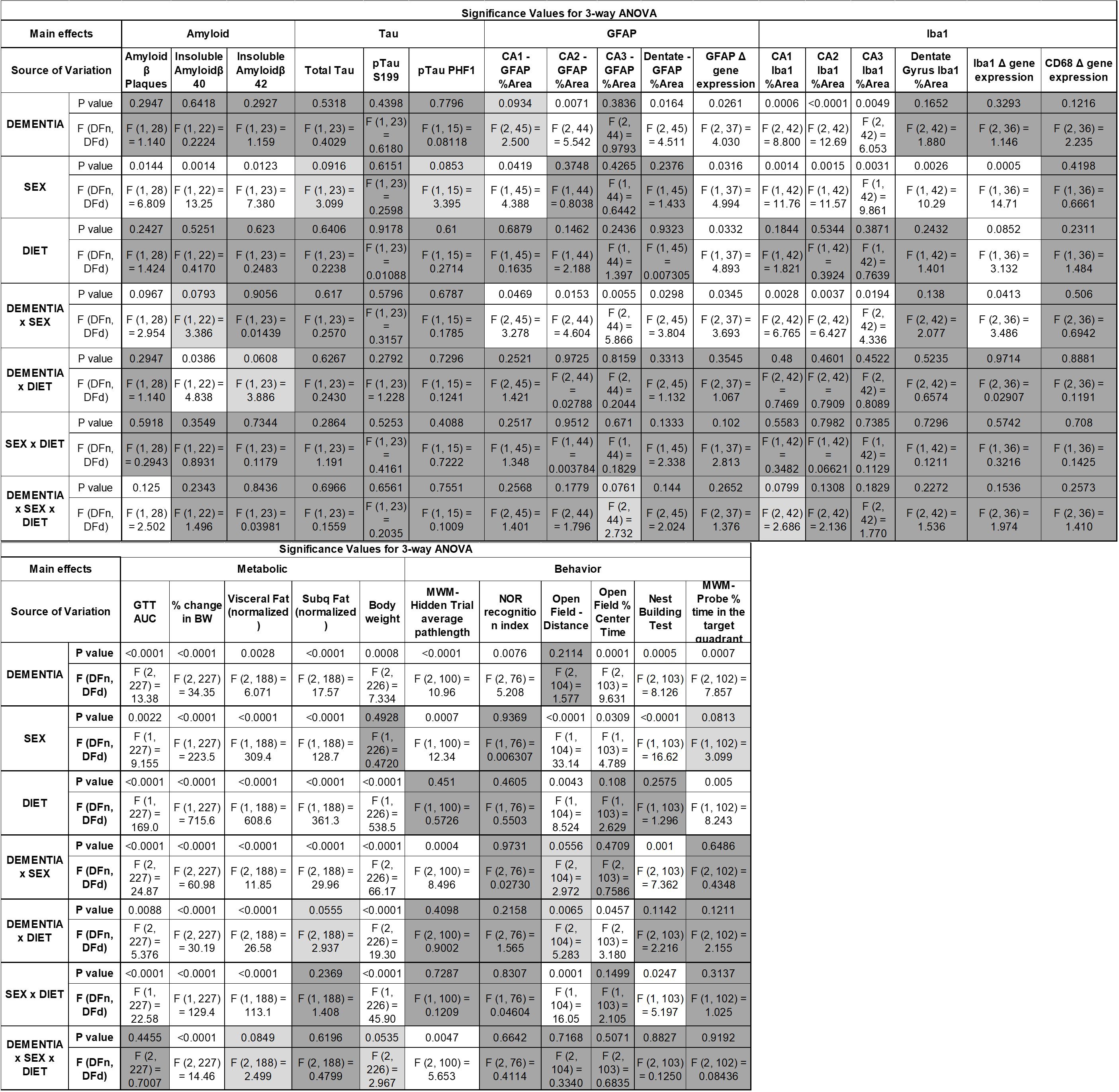

Supplement: Supplementary file 6 — Additional file 6: Table S1. Analysis of sex differences. Results (p values) of 3-way ANOVAs examining main effects of sex, diet, dementia, and interaction effects. Significant results are shown in white, values trending toward significance in light gray, and non-significant results in dark gray. [file 12974_2022_2466_MOESM6_ESM.tif]
